# Biomarkers of treatment response in rheumatoid arthritis: from conventional markers to tissue immunophenotype

**DOI:** 10.3389/fimmu.2026.1880487

**Published:** 2026-07-17

**Authors:** N.A. Batashkov, E.V. Gerasimova, D.A. Gerasimova, D.V. Svetlichnyy, E.S. Petryakina, D.I. Tychinin, S.M. Yudin, V.S. Yudin, A.A. Keskinov, V.P. Bogdanov, E.G. Zotkin, A.M. Lila, D.V. Tabakov, M. Woroncow, V.I. Skvortsova, P.Yu Volchkov

**Affiliations:** 1 Federal State Budgetary Scientific Institution “Federal Research Center for Innovator and Emerging Biomedical and Pharmaceutical Technologies”, Moscow, Russia; 2 Moscow Center for Advanced Studies, Moscow, Russia; 3 Federal State Budgetary Scientific Institution “V.A. Nasonova Research Institute of Rheumatology”, Moscow, Russia; 4 Sechenov First Moscow State Medical University, Moscow, Russia; 5 Federal State Budgetary Institution, Centre for Strategic Planning and Management of Biomedical Health Risks of the Federal Medical and Biological Agency (Centre for Strategic Planning, of the Federal Medical and Biological Agency), Moscow, Russia; 6 Russian Medical Academy of Continuous Professional Education of the Ministry of Health Care of the Russian Federation, Moscow, Russia; 7 Lomonosov Moscow State University, Moscow, Russia; 8 The Federal Medical Biological Agency (FMBA of Russia), Moscow, Russia; 9 Moscow Clinical Scientific Center N.A. A.S. Loginov, Moscow, Russia

**Keywords:** autoimmunity, immunophenotype, inflammatory, rheumatoid arthritis, treatment biomarkers

## Abstract

Rheumatoid arthritis (RA) is a biologically heterogeneous immune-mediated disease characterized by substantial variability in therapeutic response. Despite the availability of multiple conventional synthetic, biologic, and targeted synthetic disease-modifying antirheumatic drugs (DMARDs), many patients fail to achieve adequate disease control or experience secondary loss of efficacy, underscoring the need for predictive biomarkers that can guide treatment selection. This narrative review was based on a structured literature search of PubMed/MEDLINE, Scopus, Web of Science, and Google Scholar, covering publications from January 2000 to June 2026, with earlier landmark studies included when relevant. Literature selection followed PRISMA-informed principles, although the review was not designed as a formal systematic review. Unlike previous reviews that mainly catalogue RA biomarkers by analytical platform, drug class, or clinical use, this review integrates conventional and emerging biomarkers within a tissue-immunophenotype-centered framework. We critically evaluate clinical, serological, pharmacological, molecular, imaging, and tissue-based biomarkers according to biological plausibility, reproducibility, level of validation, clinical actionability, and translational readiness. Established markers such as rheumatoid factor, anti-citrullinated protein antibodies, acute-phase reactants, drug concentrations, and anti-drug antibodies remain clinically useful but provide incomplete insight into mechanism-specific therapeutic response. In contrast, synovial pathotypes, fibroblast and macrophage subsets, B-cell niches, tertiary lymphoid structures, single-cell and spatial omics, and ligand–receptor interaction networks offer a mechanistically richer view of treatment response and resistance. We conclude that precision medicine in RA will require integrated biomarker panels combining clinical, pharmacological, molecular, and synovial tissue data. The key future direction is the development of scalable, externally validated, and clinically interpretable models capable of assigning synovial endotypes and supporting mechanism-based therapeutic selection.

## 1 Introduction

Rheumatoid arthritis (RA) is a chronic systemic immune-mediated inflammatory disease characterized by persistent synovitis, progressive joint destruction, functional disability, and multiple systemic manifestations. RA affects approximately 0.5–1% of the adult population in industrialized countries and is observed two to three times more frequently in women. The incidence increases with age, with peak disease onset typically occurring in individuals over 40 years old (1). Despite substantial progress in therapeutic approaches, RA remains a progressive disease in many patients and continues to represent a major cause of reduced quality of life and long-term disability worldwide (2).

RA is highly heterogeneous, particularly at the immunological and molecular levels. Molecular profiling studies of synovial tissue and peripheral blood have demonstrated substantial variability in gene expression patterns, immune cell composition, and cytokine signaling networks between patients (3). Gene-expression analyses have identified distinct synovial pathotypes characterized by different levels of immune activation and stromal remodeling. These include lympho-myeloid, diffuse-myeloid, and pauci-immune/fibroblastic phenotypes, each associated with unique cellular compositions and inflammatory programs (4). Recent advances in single-cell transcriptomics and spatial transcriptomics have further revealed extensive heterogeneity in the synovial microenvironment, identifying diverse populations of T cells, B cells, innate lymphoid cells, macrophages, and fibroblast subsets that play distinct roles in inflammation, tissue damage, and repair (5, 6).

This biological heterogeneity has important clinical consequences. Because RA arises from multiple pathogenic mechanisms, treatment strategies are largely based on broad immunosuppressive approaches aimed at reducing inflammation and dampening immune activation. Current treatment guidelines recommend conventional synthetic disease-modifying antirheumatic drugs (csDMARDs) as first-line therapy, with methotrexate serving as the anchor drug in most treatment regimens. In patients who fail to achieve adequate disease control, therapy is typically escalated to biologic DMARDs targeting key inflammatory pathways such as tumor necrosis factor (TNF), interleukin-6 (IL-6), B cells (anti-CD20 therapy), or T-cell co-stimulation, as well as targeted synthetic DMARDs, including Janus kinase (JAK) inhibitors (7, 8).

Despite the availability of multiple therapeutic options, treatment response in RA remains highly variable. Approximately 30–40% of patients do not achieve adequate clinical response to first-line methotrexate therapy, and up to 40–50% of patients exhibit insufficient response or secondary loss of response to biologic therapies (9, 10). Currently, treatment selection is largely empirical, and reliable predictive biomarkers capable of guiding therapeutic choice are lacking. As a result, many patients undergo multiple sequential treatment switches before achieving disease control, which may lead to prolonged inflammation and irreversible joint damage.

In recent years, rapid progress in high-throughput molecular technologies—including single-cell transcriptomics, immune repertoire sequencing, spatial transcriptomics, and advanced proteomics-has significantly expanded the ability to characterize the cellular and molecular architecture of rheumatoid arthritis (5, 6). This technological shift has been accompanied by a growing number of recent review articles synthesizing emerging data on RA pathogenesis, molecular stratification, and biomarker discovery (11–13).

Several recent reviews have summarized biomarkers in RA, including traditional serological markers, pharmacological monitoring, imaging biomarkers, multi-omics approaches, and machine-learning-based precision medicine strategies. However, many of these works primarily organize biomarkers according to analytical platform, clinical use, or drug class, and therefore provide limited integration between biomarker evidence and the cellular architecture of the inflamed synovium. In recent years, in light of the development and spread of approaches to therapy aimed at the functioning of immune cells, there has been a growing interest in finding biomarkers related to the state of immune cells before and during treatment. The present review tries to consider new microenvironment biomarkers in context with other well-studied classical ones. Rather than treating biomarkers as isolated circulating analytes or descriptive molecular signatures, we emphasize how synovial pathotypes, fibroblast and macrophage subsets, B-cell niches, tertiary lymphoid structures, spatial transcriptomics, single-cell RNA sequencing, spatial proteomics, and ligand–receptor interaction networks define disease mechanisms that may influence response or resistance to specific therapies. In this framework, conventional biomarkers such as RF, ACPA, CRP, calprotectin, drug levels, and anti-drug antibodies are discussed as clinically useful but incomplete readouts, whereas tissue-based biomarkers are presented as a mechanistically richer layer for patient stratification. Thus, the novelty of this review lies in integrating classical and emerging biomarkers into a tissue-centered model of RA precision medicine, in which therapeutic selection is guided not only by systemic inflammation or serological status, but by the dominant cellular circuits operating within the synovial microenvironment.

In this context, the present review aims to address this gap by focusing specifically on biomarkers of treatment response that are supported by mechanistic insights derived from high-resolution approaches, including single-cell and immune repertoire analyses. By linking therapeutic outcomes to cellular states, immune pathways, and clonal dynamics within the synovial microenvironment, this work seeks to provide a more precise framework for biomarker development and patient stratification in RA.

In particular, emerging evidence suggests that immune phenotyping of synovial tissue, including characterization of immune cell populations, B-cell receptor and T-cell receptor repertoires, and fibroblast-like synoviocyte phenotypes, may represent especially promising directions for biomarker discovery. Understanding how these molecular and cellular features influence treatment response could enable the development of precision medicine approaches in RA.

In this review, we summarize the current state of knowledge regarding biomarkers predicting therapeutic response in rheumatoid arthritis, with a focus on both conventional and targeted therapies. We also discuss emerging technologies and conceptual frameworks that may help guide future biomarker discovery and enable more personalized treatment strategies for RA.

## 2 Methods

The literature search was conducted to identify studies investigating biomarkers of therapeutic response in rheumatoid arthritis, with particular emphasis on biomarkers supported by mechanistic evidence and studies exploring tissue-level and immune-cell–specific determinants of treatment response. The initial literature search was performed between January 2026 and May 2026. The search was last updated on 15 June 2026 to include the most recent publications relevant to synovial pathotypes, single-cell transcriptomics, spatial transcriptomics, spatial proteomics, and immune-cell interaction networks.

The following electronic databases were searched:

- PubMed/MEDLINE.
- Scopus.
- Web of Science.
- Google Scholar (for additional records and citation tracking).

Reference lists of relevant reviews and original articles were also manually screened to identify additional publications.

Search queries combined terms related to rheumatoid arthritis, biomarkers, treatment response, and specific therapeutic mechanisms. Examples of search terms included:

- “rheumatoid arthritis” AND biomarker*.
- “rheumatoid arthritis” AND treatment response.
- “predictive biomarkers” AND rheumatoid arthritis.
- “prognostic biomarkers” AND rheumatoid arthritis.
- “methotrexate” AND biomarker.
- “TNF inhibitor” AND biomarker
- “rituximab” AND biomarker.
- “tocilizumab” AND biomarker.
- “abatacept” AND biomarker.
- “JAK inhibitor” AND biomarker.
- “synovial tissue” AND rheumatoid arthritis.
- “synovial pathotype”.
- “single-cell transcriptomics” AND rheumatoid arthritis.
- “immune repertoire” OR “AIRR-seq” AND rheumatoid arthritis.
- “precision medicine” AND rheumatoid arthritis.

The primary search included studies published between January 2000 and March 2025.

Earlier landmark studies were included when they represented foundational work on established biomarkers (for example methotrexate pharmacogenetics).

Studies were considered eligible if they follow at least one of these inclusion criteria:

- investigated biomarkers associated with therapeutic response, resistance, disease activity, or prognosis in RA;
- evaluated conventional synthetic, biologic, or targeted synthetic DMARDs;
- included clinical, serological, pharmacological, molecular, transcriptomic, proteomic, imaging, or tissue-based biomarkers;
- provided mechanistic insight into biomarker function or biological relevance;
- were original research articles, meta-analyses, systematic reviews, or high-quality narrative reviews;
- were published in peer-reviewed journals;
- were available in English.

Studies were excluded if they:

- did not focus on rheumatoid arthritis;
- investigated biomarkers unrelated to disease activity or therapeutic outcomes;
- were conference abstracts without full-text publication;
- were case reports or very small exploratory studies lacking sufficient methodological detail;
- contained insufficient clinical or biological data for interpretation;
- were duplicate publications.

Titles and abstracts retrieved through the database search were screened for relevance. Full-text articles were subsequently evaluated for eligibility according to the predefined inclusion and exclusion criteria.

Particular attention was given to studies providing:

- mechanistic evidence linking biomarkers to specific therapeutic pathways;
- validation in independent cohorts;
- prospective evaluation of predictive performance;
- tissue-based, single-cell, or immune-repertoire analyses.

Priority was assigned to meta-analyses, systematic reviews, prospective cohorts, randomized clinical trials, and large multicenter studies.

This review was designed as a narrative review with a structured literature search rather than a formal systematic review. Therefore, a full PRISMA flow diagram was not generated. However, literature identification, screening, eligibility assessment, and study selection were performed according to PRISMA-informed principles to improve transparency and minimize selection bias.

## 2 Classification of biomarkers type

Biomarkers represent key tools of precision medicine, enabling the characterization of disease processes at the biological level. A biomarker is defined as a measurable indicator of normal biological processes, pathogenic mechanisms, or pharmacological responses to therapeutic interventions (14).

In RA, biomarkers are most usefully classified according to their clinical function, particularly their ability to inform prognosis or guide therapeutic decision-making. From this perspective, biomarkers can be broadly divided into prognostic and predictive categories.

**Figure 1:**
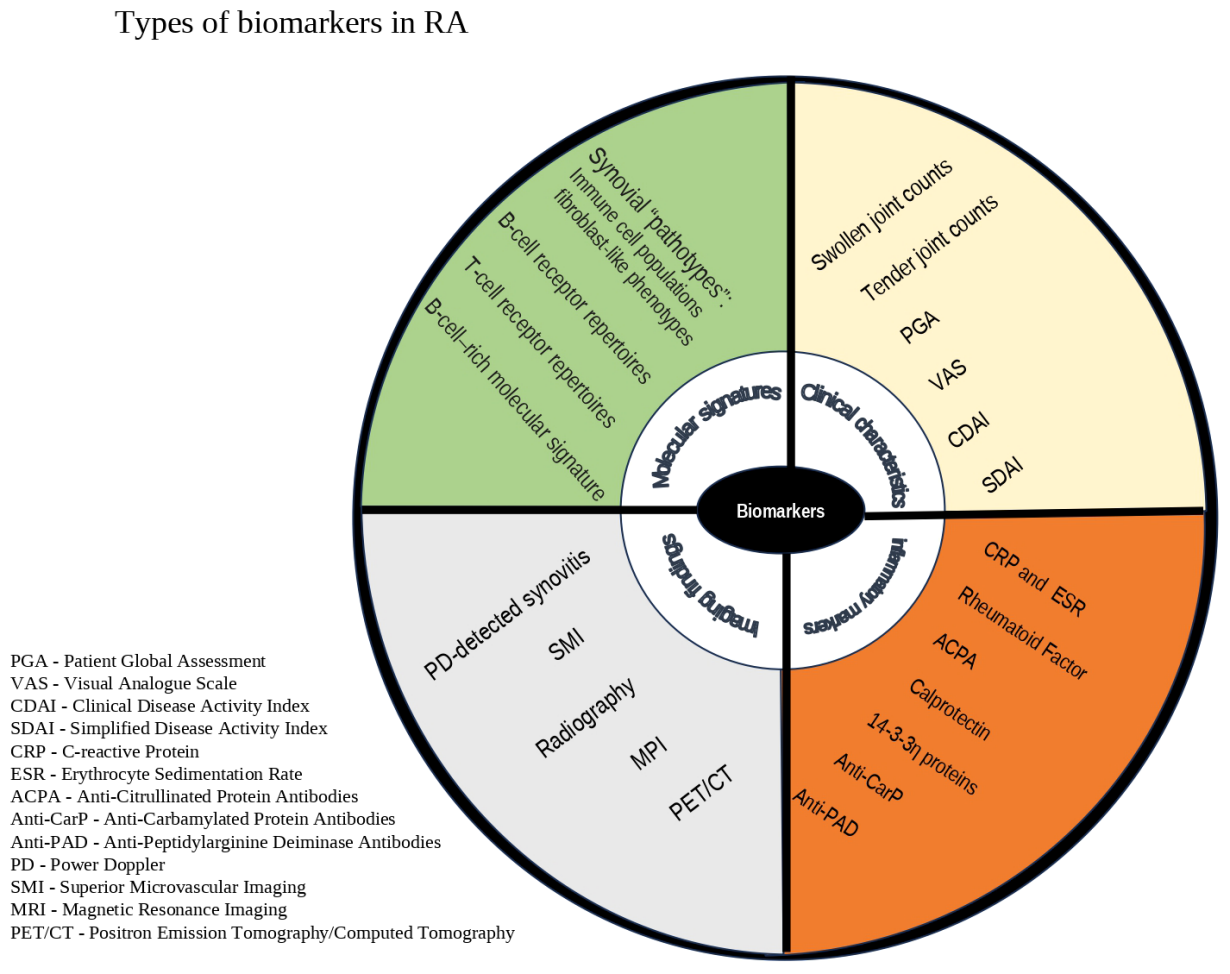
Known biomarkers for rheumatoid arthritis treatment.

Prognostic biomarkers provide information about the expected course or outcome of disease independently of treatment. In RA, such markers may include clinical characteristics, serological autoantibodies, inflammatory markers, imaging findings, and molecular signatures associated with structural progression or functional decline (9). These biomarkers help identify patients at higher risk of aggressive disease and may influence treatment intensity or monitoring strategies (**Figure 1**).

In contrast, predictive biomarkers are used to estimate the likelihood of response to a specific therapeutic intervention. Predictive biomarkers are of particular importance in RA because treatment responses vary widely even among patients receiving the same drug. These markers often reflect the activity of specific pathogenic pathways targeted by therapy, such as TNF-driven inflammation, B-cell–mediated autoimmunity, T-cell activation, or IL-6 signaling. Identification of reliable predictive biomarkers is therefore essential for improving treatment stratification and advancing precision medicine approaches in RA.

**Figure 2:**
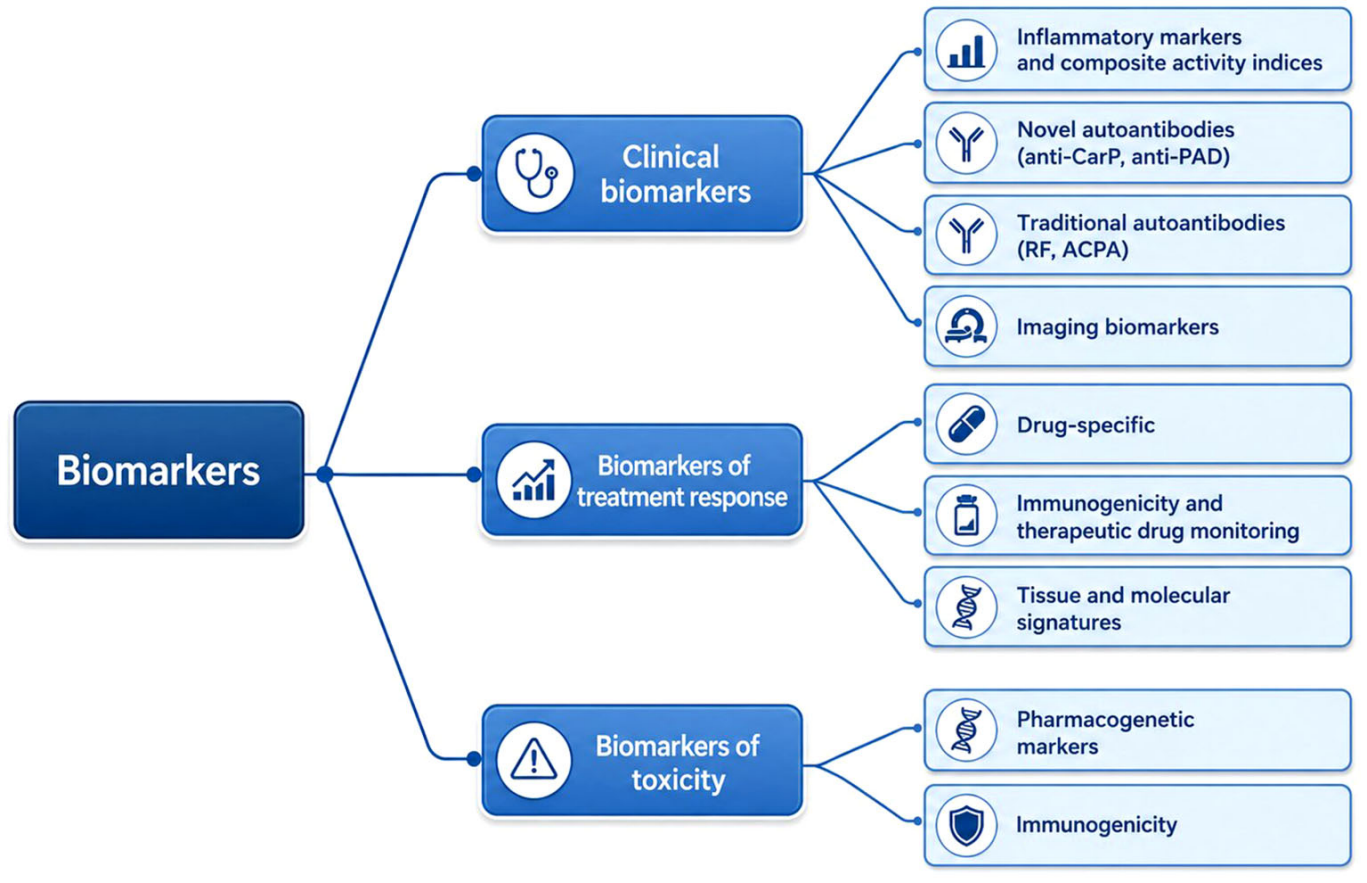
Biomarkers classification in RA.

Within the predictive biomarker category, further subclassification can be made based on the type of biological information they provide. This includes clinical biomarkers, pharmacological biomarkers, molecular biomarkers, and imaging biomarkers reflecting synovial inflammation. Each of these biomarker types offers distinct advantages and limitations in terms of feasibility, specificity, and clinical applicability (**Figure 2**).

For biomarkers to be clinically useful, they must be standardized, reproducible, and actionable, meaning that their measurement should lead to meaningful changes in clinical decision-making, such as treatment selection, dose optimization, or toxicity prevention (15).

In the following sections, we review current evidence on biomarkers of therapeutic response in RA, structured according to their clinical relevance and biological level, including clinical, pharmacological, molecular, and imaging predictors of response. This framework is aligned with widely accepted regulatory classifications proposed by the U.S. Food and Drug Administration and European Medicines Agency, which categorize biomarkers based on their clinical function, including diagnostic, prognostic, predictive, monitoring, pharmacodynamic, and safety biomarkers. Within this framework, predictive and prognostic biomarkers represent key categories for precision medicine in RA, providing the conceptual basis for treatment stratification and outcome assessment. These definitions are further formalized in the BEST (Biomarkers, EndpointS, and other Tools) resource developed by the FDA-NIH Biomarker Working Group.

### 2.1 Clinical immune biomarkers

Clinical biomarkers are routinely collected measures that quantify current inflammatory burden, functional impact, and structural risk. They are widely used because of accessibility and established thresholds. In RA, clinical “tools” are used to indirectly assess signs of inflammation - pain and swelling of the joints, but not the actual intensity of the inflammatory lesion of the synovial membrane and internal organs. Calculating the painful and swollen of the joints is characterized by relatively low reproducibility, does not allow for the detection of subclinical joint inflammation, and does not take into account other possible manifestations of rheumatoid inflammation (tenosynovitis, fibromyalgia, etc.) (16).

#### 2.1.1 Inflammatory markers and composite activity indices

Disease activity in rheumatoid arthritis is commonly assessed using composite clinical indices. One of the most widely used is the Disease Activity Score (DAS), particularly its modified version DAS28, which integrates the number of tender and swollen joints (out of 28 assessed joints), the erythrocyte sedimentation rate (ESR) or C-reactive protein (CRP) levels as markers of systemic inflammation, and the patient’s global assessment (PGA) of disease activity. However, DAS values are highly dependent on acute-phase reactants, particularly ESR and CRP levels. This may lead to overestimation of therapeutic effectiveness for certain drugs, such as interleukin-6 (IL-6) inhibitors and Janus kinase (JAK) inhibitors, which exert a strong pharmacological effect on acute-phase markers and therefore reduce ESR and CRP more profoundly than some clinical parameters, such as the number of tender or swollen joints and the patient global assessment. As a result, improvements in DAS scores in patients receiving these therapies may partly reflect changes in laboratory markers rather than true resolution of synovial inflammation (17).

Because of this potential bias, the American College of Rheumatology/European League Against Rheumatism (ACR/EULAR) recommendations currently emphasize the use of the Simplified Disease Activity Index (SDAI) and Boolean remission criteria for defining remission in rheumatoid arthritis (18). These indices differ from DAS-based measures in both their structure and sensitivity to inflammatory versus non-inflammatory disease components.

The Boolean remission criteria are even more stringent and require that all key disease activity parameters simultaneously fall below predefined thresholds (tender joint count ≤1, swollen joint count ≤1, CRP ≤1 mg/dL, and patient global assessment ≤1 on a 0–10 scale). This approach minimizes the risk that improvement in a single parameter—such as CRP suppression—will falsely indicate remission.

Another important aspect of the problem with measuring disease activity concerns the contribution of patient-reported outcomes, particularly the PGA, which is a key component of all major RA disease activity indices. PGA represents the patient’s own assessment of overall disease activity, typically measured using a visual analogue scale (VAS) ranging from 0–10 cm (or 0–100 mm).

However, PGA values may be influenced by multiple factors unrelated to active synovial inflammation, including chronic pain, fatigue, functional disability, psychological factors, and comorbid musculoskeletal conditions such as fibromyalgia or osteoarthritis (19). Approximately one-third of RA patients who have no tender or swollen joints on clinical examination and CRP ≤1 mg/L still report PGA values >1, indicating persistent patient-reported disease burden despite minimal objective inflammatory activity (20). This phenomenon has led to the concept of the “PGA-driven remission paradox”.

Also, there are several inflammatory markers to help physicians.

CRP is an acute phase protein, which is produced by the liver in response to inflammatory stimuli. In RA, the production of pro-inflammatory cytokines, particularly interleukin-6 (IL-6), leads to an elevation of CRP levels (21). CRP and ESR remain core laboratory biomarkers integrated into treat-to-target strategies through composite indices such as DAS28 (22). However, the choice of CRP- vs ESR-based indices can shift classification of disease activity and remission, impacting treatment decisions and interpretation of response. CRP monitoring has potential utility for prognostic assessment and treatment guidance in RA. Higher baseline CRP levels have been linked to greater radiographic joint damage and disability in diverse patient populations (23). There is evidence that CRP levels >7.1 mg/L predicted poor outcome with traditional synthetic disease-modifying antirheumatic drugs (DMARDs) (24).

Recent biomarker-focused studies still place CRP/ESR among the most commonly used measures for monitoring and prognosis, while emphasizing their limitations (non-specificity; confounding by comorbid inflammation, age, etc.) (25).

Calprotectin (also known as S100A8/F9 and MRP8/14) is attracting attention as a promising biomarker. Calprotectin is one of the most consistently replicated inflammatory biomarkers in RA and appears biologically attractive because it reflects local myeloid-cell activation rather than hepatic acute-phase responses (26).

Calprotectin has been proposed as a biomarker for monitoring, predicting erosion progression and therapeutic response, especially in patients receiving effective biological treatment (e.g., TNF-α inhibitors and IL-6 inhibitors) (27, 28). However, despite superior correlations with synovitis in some studies compared with CRP, uncertainty remains regarding clinically meaningful cutoffs, assay standardization, and incremental value over existing composite disease activity measures. Therefore, calprotectin currently represents a promising monitoring biomarker but cannot yet be considered a validated predictive biomarker for treatment selection.

Another promising biomarker is 14-3-3η proteins. The concentration of the 14-3-3η isoform in the synovial fluid and blood serum of patients with RA is higher than in healthy individuals (29). The 14-3-3η protein accumulates in the cytoplasm of synovial tissue macrophages and then enters the extracellular space after necroptosis mediated by the action of TNF-α (30). It has been noted that 14-3-3η, like TNF-α, is capable of inducing the synthesis of matrix metalloproteinases (MMPs) by activating ERK (extracellular signal-regulated kinase) and JNK/SAPK (c-Jun N-terminal kinases/stress-activated protein kinase).There is evidence that JNK hyperactivation correlates with the progression of undifferentiated arthritis to RA, and the level of ERK phosphorylation is a predictor of the rapid development of erosions (31). In the early stages of RA, determining 14-3-3η in the serum of patients will allow one to assess the likelihood of developing erosions and osteoporosis, and in the advanced stage, to predict the effectiveness of treatment (32) It has been established that an increase in the concentration of 14-3-3η in patients with RA can be a predictor of a good “response” to therapy with an IL-6 inhibitor (tocilizumab) and TNF-α inhibitors (33, 34).

While cytokine assays (TNF-α, IL-6, IL-17) and B-cell phenotyping (plasmablast frequency, memory B-cell subsets) provide insight into the pathogenesis of RA, their use in routine clinical practice remains limited. Measurements of individual cytokines lack the necessary specificity and reproducibility for patient-centered decision-making. The complexity and variability of their serum concentrations hinder their use outside of specialized research protocols.

The multi-biomarker disease activity (MBDA) test is a commercially CLIA-certified serum multi-marker that includes 12 protein analytes relevant to RA: inflammation epidermal growth factor (EGF); IL-6; matrix metalloproteinase 1 and 3 (MMP-1 and MMP-3); serum amyloid A (SAA); TNF receptor 1 (TNF-R1); vascular cell adhesion molecule 1 (VCAM-1); vascular endothelial growth factor-1 (VEGF-1); chitinase-3-like protein 1 (YKL-40); CRP; leptin; and resistin]. These 12 analytes were derived from an initial candidate panel of 130 circulating proteins using samples from multiple RA cohorts from North America and Europe (35). It was correlated with disease activity (DAS28), arterial inflammation and was shown to be predictive of future radiographic damage (36). MBDA scores correlated with subsequent treatment response, predicting remission within 1 year (24).

#### 2.1.2 Traditional autoantibodies: anti-citrullinated protein antibodies and rheumatoid factor

RA can be broadly divided into two major clinical and immunological subtypes based on the presence of rheumatoid factor (RF) and anti-citrullinated protein antibodies (ACPA). These subtypes differ substantially in their molecular pathogenesis, genetic risk factors, environmental triggers, and clinical course. In particular, ACPA-positive RA is strongly associated with HLA-DRB1 shared epitope alleles, whereas ACPA-negative disease demonstrates weaker or different genetic associations (37). Environmental factors such as smoking, periodontitis, and pulmonary inflammation also appear to preferentially contribute to the development of the ACPA-positive subtype (38).

The production of autoantibodies is one of the earliest detectable events in RA pathogenesis. RF and ACPA may be present several years before the onset of clinical arthritis and therefore represent important tools for early disease classification and stratification. Clinically, these antibodies are routinely measured not only for diagnostic purposes but also for disease stratification and assessment of prognosis. At the immunological level, the two major serological subtypes of RA are characterized by distinct inflammatory mechanisms. ACPA-positive RA is generally associated with stronger activation of the adaptive immune system, including enhanced B-cell responses and T-cell–mediated autoimmunity. Synovial tissue from seropositive patients often demonstrates increased infiltration of CXCL13-producing CD4^+^ T cells, which contribute to B-cell recruitment and the formation of ectopic lymphoid structures within the inflamed synovium. In contrast, ACPA-negative RA tends to exhibit weaker adaptive immune signatures and a relatively greater contribution of innate immune pathways to disease pathogenesis (39).

These biological differences are reflected in clinically meaningful outcomes. High levels of ACPA, particularly when combined with RF positivity, are consistently associated with a more severe disease phenotype, characterized by higher inflammatory activity, accelerated radiographic progression, increased frequency of extra-articular manifestations, and elevated long-term mortality risk. RF positivity alone has also been linked to a more aggressive disease course, including higher baseline disease activity and greater likelihood of structural joint damage, although the strongest prognostic signal is typically observed in patients with double seropositivity, RF and ACPA. Together, these findings support the concept that serological status reflects underlying immunopathogenic mechanisms that shape both disease severity and therapeutic responsiveness in RA.

Conversely, differences in underlying immune mechanisms between serological subtypes may also influence responses to other targeted therapies. For instance, the relatively stronger contribution of innate immune pathways in ACPA-negative RA has been proposed as a possible explanation for the lower efficacy of T-cell costimulation blockade with abatacept observed in this subgroup compared with ACPA-positive disease. Together, these observations illustrate how classical serological markers, originally developed for diagnosis, can also provide clinically meaningful insights into disease biology and therapeutic stratification.

#### 2.1.3 Novel autoantibodies

Along with ACPA, which are antibodies to the products of the enzymatic conversion of arginine to citrulline, attention has been drawn to Anti-Carbamylated Protein Antibodies (Anti-CarP). According to experimental studies, the development of an immune response to carbamylated proteins was accompanied not only by the synthesis of anti-CarbP, but also by interferon-γ (IFN-γ), IL-10 and IL-17, chemotaxis and proliferation of CD4+ T-lymphocytes associated with the development of erosive arthritis (40).

Of great significance is the fact that anti-CarbP are detected in 8–24% of cases in ACPA-negative patients, and therefore are important for the early diagnosis of RA (41, 42). An association has been noted between the detection of anti-CarbP and the development of bone erosions, especially in ACPA-negative patients (43, 44). Furthermore, in anti-CarbP-positive patients, compared with negative ones, regardless of the detection of ACPA, higher inflammatory activity indices (ESR, CRP) and DAS28 values ​​were observed. It is noteworthy that an increase in anti-CarbP levels is associated with interstitial lung disease (45) and the development of subclinical atherosclerotic vascular lesions (46).

As is known, ACPA-positive RA patients respond better to abatacept therapy; also, effective treatment with abatacept was associated with the detection of anti-CarbP and was accompanied by a decrease in autoantibody titers (47). It is proposed to conduct protein testing to confirm RA in patients seronegative for RF and ACPA, as well as in seropositive patients for whom additional prognostic stratification would influence treatment intensity decisions (32). However, this approach is not currently supported by guidelines.

Peptidylarginine Deiminase (PAD) enzymes catalyze the conversion of arginine to citrulline. PAD4 and cross-reactive anti-PAD3/4 antibodies associated with aggressive disease phenotypes (1). Anti-PAD3/4 potentially useful for prognostic stratification in ACPA-positive patients, particularly for identifying patients at the highest risk of aggressive, erosive disease or interstitial lung disease (48). RA patients who have antibodies to PAD4 have significant joint damage, but they respond favorably to tofacitinib therapy when methotrexate monotherapy is ineffective (49).

Clinical application: They are potentially useful in ACPA seronegative patients, particularly for determining the risk of aggressive erosive disease or extra-articular manifestations. Standardized commercial assays for detecting new antibodies are emerging, but they have not yet been validated or widely adopted.

#### 2.1.4 Imaging biomarkers

Musculoskeletal ultrasound has emerged as one of the most informative imaging tools for assessing inflammatory activity in RA. Compared with standard clinical examination, ultrasound provides higher sensitivity for detecting synovitis, including subclinical inflammation that may not be apparent during joint palpation. As a result, imaging biomarkers derived from ultrasound—particularly those reflecting synovial vascularity and inflammatory activity—have attracted increasing attention as objective indicators of disease activity and treatment response.

One of the most widely studied ultrasound biomarkers is Power Doppler (PD) signal, which detects blood flow within the synovial tissue and therefore serves as an indirect marker of active synovial inflammation and angiogenesis. PD positivity reflects increased vascularization of the inflamed synovium and correlates with histological markers of inflammatory infiltration. Several studies have evaluated PD signal as a potential predictive biomarker of treatment response, particularly in the context of treatment escalation. A recent systematic synthesis reported that the presence of PD-positive synovitis may help identify patients more likely to benefit from intensification of therapy (50). In this context, PD functions primarily as a predictive biomarker of response, indicating the presence of active inflammatory processes that may still respond to immunosuppressive therapy.

In addition to predicting treatment response, PD ultrasound has also been investigated as a prognostic biomarker of disease progression. Longitudinal studies have demonstrated that persistence of PD-detected synovitis is associated with poorer clinical outcomes, including increased risk of radiographic progression and disease relapse. Importantly, PD activity may remain detectable even when patients achieve apparent clinical remission according to composite disease activity indices. This phenomenon highlights the presence of subclinical synovitis, which may contribute to ongoing joint damage despite apparently controlled disease activity (51). Thus, PD ultrasound may function as a biomarker of residual inflammatory activity that is not captured by clinical scoring systems.

Despite these advantages, several limitations restrict the widespread use of PD as a standardized biomarker. Ultrasound assessment is operator-dependent, and the sensitivity of PD signal detection can vary depending on equipment settings, probe frequency, and scanning technique. In addition, there is currently no universally accepted threshold for clinically meaningful PD positivity, and scoring systems may differ across studies and clinical centers. These methodological differences contribute to variability in reported predictive performance and complicate the integration of PD findings into routine treatment algorithms.

More recently, novel ultrasound technologies have been introduced to improve detection of low-flow microvascular signals within the synovium. One such method is superior microvascular imaging (SMI), which uses adaptive algorithms to separate slow blood-flow signals from tissue motion artifacts. This approach allows more sensitive visualization of microvascular perfusion within small synovial vessels, potentially providing a more precise assessment of inflammatory angiogenesis. A meta-analysis comparing SMI with conventional PD demonstrated that SMI had higher sensitivity for detecting synovial blood flow across different disease activity levels, with a pooled odds ratio of 2.12 (95% CI 1.80–2.51, P < 0.01) for detecting vascular signals (52). Furthermore, studies applying the OMERACT-EULAR ultrasound scoring system have confirmed that SMI can detect synovial vascularization with greater sensitivity than PD while maintaining comparable scoring reliability (53).

However, despite its improved sensitivity, SMI also faces several challenges. Increased sensitivity may lead to detection of physiological microvascular signals that are not necessarily associated with active inflammation, potentially reducing specificity. Moreover, SMI technology is not yet widely available in clinical ultrasound platforms, and standardized scoring criteria comparable to those established for PD are still under development.

Overall, ultrasound-based biomarkers illustrate the growing importance of imaging-derived indicators of synovial inflammation in RA. Power Doppler signal currently represents the most established imaging biomarker, functioning primarily as an indicator of active and residual inflammation as well as potential treatment responsiveness. Emerging techniques such as SMI may further improve the detection of microvascular inflammation, although additional studies are needed to determine their clinical specificity, standardization, and prognostic value in routine RA management.

Conventional radiography is generally used to depict erosions in RA. Magnetic resonance imaging (MRI) has shown its value by visualizing (subclinical) joint inflammation and thereby identifying patients at high risk of progression to RA (54).

The use of MRI is limited by cost and analysis time, but the use of MRI in clinical practice is expected to increase due to the development of short scanning protocols (such as the modified Dixon sequence) and the use of artificial intelligence (55). Joint inflammation detected by MRI is also a component of the EULAR/ACR risk stratification criteria for the development of RA (56, 57).

In a recent large observational study comparing two imaging modalities (radiography and MRI), MRI-detected erosions in patients with symptomatic arthralgia at risk for RA developing, including those who were ACPA-positive, were of no significant significance and were not associated with radiographic progression (57).

Among imaging biomarkers, power Doppler ultrasound currently has the strongest evidence base because it repeatedly demonstrates associations with residual inflammation, radiographic progression, and treatment response. However, its predictive performance is highly operator-dependent and varies according to equipment settings and scoring systems. Emerging technologies such as SMI may improve sensitivity, but whether increased sensitivity translates into superior clinical decision-making remains unclear. Consequently, imaging biomarkers are currently more useful for monitoring residual inflammation than for selecting specific therapeutic mechanisms.

Molecular imaging techniques that allow for the earlier diagnosis of RA and the quantification of inflammation and complications. Positron emission tomography/computed tomography (PET/CT) with [18F]-fluorodeoxyglucose ([18F]-FDG) and other radioactive tracers is now increasingly used to diagnose and monitor RA.

In recent years, molecular imaging tracers have been actively studied for detecting or monitoring RA activity, with a focus on adhesion molecules (vascular adhesion protein-1), macrophages (translocator protein (TSPO) in activated macrophages), and fibroblast-like synoviocytes (fibroblast activation protein inhibitor (FAPI)).With the introduction of PET/CT, it has become possible to diagnose or even predict future complications of RA (vasculitis, rheumatoid nodules, interstitial lung disease, cardiovascular diseases, neurological, nephrological, ophthalmological and hematological complications (58).

The use of FAPI-enhanced PET/CT appears to be a promising approach. The theranostic potential of FAPI has led to the development of a new treatment option for RA. One study demonstrated the use of FAP-targeted photodynamic therapy as a targeted local therapy for RA (59).

### 2.2 Biomarkers of treatment response

In the context of RA, the most significant interest lies in predictive biomarkers that can forecast the effectiveness of a particular treatment. In this section, we summarize the attempts made in recent years to identify reliable biomarkers of treatment response.

#### 2.2.1 Drug-specific

The effectiveness of drugs in the treatment of RA varies significantly among patients due to genetic, immunological, and metabolic heterogeneity. Drug-specific biomarkers (specific metabolites or components of a biochemical pathway associated with drug response) hold tremendous potential for optimizing treatment strategies. The integration of drug-specific biomarkers into clinical practice empowers clinicians to predict treatment response, identify high-risk patients for adverse effects, and optimize individual dosing regimens, thereby maximizing efficacy and minimizing toxicity.

One of the best-known examples is methotrexate polyglutamates (MTX-PG). Intracellular MTX-PG have been extensively studied as pharmacological biomarkers potentially associated with both treatment efficacy and toxicity. Methotrexate (MTX) is metabolized intracellularly through the sequential addition of glutamate residues by folylpolyglutamate synthase, forming MTX-PGs, which accumulate in red blood cells and serve as a marker of the drug’s intracellular effect. MTX-PG concentrations in red blood cells were quantified using liquid chromatography-tandem mass spectrometry (LC-MS/MS), which allowed for the measurement of individual polyglutamate species (60). The researchers applied population pharmacokinetic-pharmacodynamic modeling in patients initiating MTX therapy for RA to investigate the relationships between MTX-PG exposure and clinical outcomes. The analysis showed that higher cumulative MTX-PG exposure was associated with an improved clinical response, as assessed by a reduction in disease activity indices.

In real-world clinical practice, the implementation of drug-specific biomarkers provides clinical and economic advantages through the avoidance of ineffective therapy. Moreover, the use of the biomarkers to guide individualized dosing regimens reduces the risk of drug overdose and associated toxicities. This, in turn, lowers the costs of complication management, enhances patients’ quality of life, and contributes to reduced disability rates and long-term healthcare burdens.

Nevertheless, the clinical and economic value of drug-specific biomarkers remains debatable. Although MTX-PG levels have shown promising results as guides for MTX treatment, their broader implementation is hampered by interindividual variability, technical limitations, and the need for standardized assays. Comorbidities and concomitant medication use can affect drug metabolism and efficacy, further complicating the interpretation of biomarkers. Additionally, the situation is aggravated by the necessity for considerable initial investments in the biomarkers research, development, and clinical implementation, along with elevated costs of the initial diagnostic work-up required for patient assessment.

Drug-specific biomarkers have considerable potential to improve treatment outcomes and reduce healthcare costs through personalized therapy, prevention of adverse events, and optimized resource utilization. However, realizing this potential is contingent upon several critical factors: the affordability of biomarker testing, the robust clinical validity of the biomarkers, the establishment of clear clinical protocols for their use, the adequacy of healthcare infrastructure, and the system’s capacity to integrate novel technologies effectively.

#### 2.2.2 Immunogenicity and therapeutic drug monitoring

For monoclonal antibodies and other biologic DMARDs, pharmacological biomarkers of treatment response commonly include trough drug concentrations and anti-drug antibodies (ADA). These parameters are used within the framework of therapeutic drug monitoring (TDM) to evaluate drug exposure and identify pharmacokinetic causes of treatment failure (61). Trough drug levels are typically measured immediately before the next scheduled dose, providing an estimate of minimal circulating drug concentration during the dosing interval. Measurement of both trough levels and ADA allows differentiation between pharmacokinetic failure, caused by insufficient drug exposure (often due to immunogenicity), and pharmacodynamic failure, where adequate drug levels are present but the patient does not respond due to alternative pathogenic mechanisms.

Drug concentrations and ADA are most commonly quantified using immunoassay-based techniques, including enzyme-linked immunosorbent assays (ELISA), radioimmunoassays, electrochemiluminescence assays, and more recently bridging immunoassays or drug-tolerant assays capable of detecting antibodies in the presence of circulating drug. In some research settings, liquid chromatography–mass spectrometry (LC-MS/MS) methods are also used for precise quantification of biologic drug concentrations. Among these, ELISA-based assays remain the most widely implemented due to their relative simplicity and cost-effectiveness.

In clinical practice, TDM has been studied most extensively for TNF inhibitors, including infliximab, adalimumab, and etanercept. For example, multiple studies have demonstrated that low trough levels of infliximab or adalimumab are associated with reduced therapeutic response, whereas the presence of ADA is frequently linked to accelerated drug clearance and secondary loss of response. Conversely, patients with adequate drug concentrations but persistent disease activity are more likely to benefit from switching to a therapy with a different mechanism of action rather than increasing the dose of the same drug. These principles have been incorporated into several proposed TDM-based treatment algorithms for TNF inhibitor therapy.

Despite these promising applications, the routine use of drug-level monitoring in RA remains controversial. Some clinical trials and observational studies suggest that TDM-guided dose adjustment may improve treatment efficiency and reduce unnecessary switching, whereas others have found limited additional clinical benefit compared with standard treat-to-target strategies (62). Moreover, several factors limit widespread implementation in routine rheumatology practice. These include variability between assay platforms, lack of universally accepted therapeutic thresholds, differences in pharmacokinetics between biologic agents, and uncertainty regarding the optimal timing and interpretation of measurements. As a result, while TDM is increasingly used in certain clinical contexts-particularly in cases of secondary loss of response or suspected immunogenicity - it has not yet become a standard component of RA management guidelines.

A large prospective cohort analysis reported an association between ADA and nonresponse to biologic drugs in RA, supporting clinical interest in monitoring- particularly among nonresponders (7).

#### 2.2.3 Tissue and molecular signatures (synovial precision biomarkers).

The strongest signals for predictive biomarkers in RA increasingly come from studies of synovial tissue biology, which directly reflects the inflammatory microenvironment driving disease activity. Unlike peripheral blood biomarkers, synovial tissue analysis provides information about the local cellular composition, molecular signaling pathways, and immune interactions within the joint, which are central to RA pathogenesis. As a result, several studies have demonstrated that molecular and cellular signatures within the synovium can stratify patients into biologically distinct disease subsets with different therapeutic responses.

Taken together, these cellular frameworks strengthen the central thesis that RA biomarker discovery is shifting from circulating single-analyte predictors toward tissue-level immunophenotyping. Synovial pathotypes provide the first layer of classification; fibroblast and macrophage subsets refine the stromal and innate immune components; B-cell niches and TLS define local adaptive immune organization; and single-cell, spatial transcriptomic, spatial proteomic, and ligand–receptor analyses reconstruct the cellular circuits that drive inflammation and treatment response. This integrated tissue-immunophenotype framework better matches the biological complexity of RA and provides a stronger rationale for mechanism-based therapeutic selection than conventional biomarkers alone.

##### 2.2.3.1 Synovial pathotypes as tissue-level biomarkers of therapeutic response

Synovial pathotypes represent one of the most biologically grounded approaches to patient stratification in rheumatoid arthritis, because they classify patients according to the cellular and molecular organization of the inflamed target tissue rather than by peripheral blood markers alone. Histological and transcriptomic studies of synovial biopsies have reproducibly identified major tissue patterns, most commonly described as lympho-myeloid, diffuse-myeloid, and pauci-immune/fibroblastic pathotypes. These patterns differ in immune-cell composition, stromal activation, inflammatory mediators, and association with treatment response, making them attractive candidates for mechanism-based biomarker development. The PEAC cohort provided key evidence that synovial cellular and molecular signatures can stratify response to csDMARD therapy and predict radiographic progression in early RA (63). Further transcriptomic profiling of early RA synovium demonstrated that these molecular portraits are associated with clinical phenotypes and treatment-response patterns (64).

###### 2.2.3.1.1 Lympho-myeloid pathotype

The lympho-myeloid pathotype is characterized by dense infiltration of adaptive immune cells, including B cells, T cells, plasma cells, and myeloid cells. This subtype frequently shows high expression of genes associated with antigen presentation and adaptive immune activation, including HLA class II molecules, immunoglobulin transcripts, B-cell lineage markers, and chemokines such as CXCL13. CXCL13 is particularly important because it promotes B-cell recruitment and organization within inflamed synovial tissue and is closely linked to the formation of tertiary lymphoid structures (TLS). TLS-like aggregates in RA synovium may support local antigen presentation, B-cell maturation, plasma-cell differentiation, and potentially local autoantibody production, thereby sustaining chronic inflammation.

From a biomarker perspective, the lympho-myeloid pathotype is important because it indicates a disease state in which adaptive immune mechanisms and B-cell–driven inflammation is dominant. This provides a mechanistic rationale for improved response to B-cell–targeted therapy, particularly rituximab. In R4RA, a biopsy-driven randomized trial in TNF inhibitor inadequate responders, patients with low synovial B-cell molecular signatures had markedly lower response to rituximab than to tocilizumab, whereas patients with higher B-cell lineage signatures appeared more suitable for B-cell depletion strategies (6, 65). Thus, the lympho-myeloid pathotype is not merely a descriptive tissue phenotype, but a potential predictive biomarker indicating the presence of the therapeutic target and a humoral immune program within the synovium.

###### 2.2.3.1.2 Diffuse-myeloid pathotype

The diffuse-myeloid pathotype is characterized by prominent infiltration of macrophages and other myeloid-lineage cells, but without the highly organized lymphoid aggregates typical of the lympho-myeloid subtype. This pathotype is enriched for inflammatory programs related to innate immune activation, including cytokine networks involving TNF, IL-1, and IL-6. The predominance of macrophage-derived inflammatory mediators suggests that this subtype may be driven more by innate immune amplification than by organized local humoral immunity.

Therapeutically, the diffuse-myeloid pathotype may be relevant for therapies targeting inflammatory cytokine circuits, including TNF inhibitors and IL-6 pathway blockade. However, its predictive value is less clearly established than that of the B-cell–rich lympho-myeloid pathotype for rituximab. In early RA cohorts, myeloid-rich synovial signatures have been associated with active inflammation and clinically relevant disease phenotypes, but their ability to select one specific cytokine-targeted therapy over another remains incompletely validated (63, 64). Therefore, the diffuse-myeloid pathotype should currently be interpreted as a biologically meaningful inflammatory endotype, but not yet as a fully validated treatment-selection biomarker.

###### 2.2.3.1.3 Pauci-immune pathotype

The pauci-immune pathotype or fibroblastic pathotype is defined by relatively low infiltration of immune cells and relative dominance of stromal and fibroblast-like synoviocyte programs. Compared with lympho-myeloid and diffuse-myeloid synovitis, this subtype shows reduced expression of immune-cell markers, lower lymphoid organization, and stronger representation of tissue-remodeling, extracellular matrix, and fibroblast-associated pathways. Clinically, this is particularly important because inflammatory burden measured in blood may be relatively modest, whereas symptoms such as pain and fatigue may remain substantial.

From the perspective of therapeutic response, the pauci-immune/fibroblastic pathotype may represent a state in which conventional cytokine- or lymphocyte-targeted biologics are less effective, because the dominant tissue program is not primarily driven by dense immune-cell infiltration. In a study of TNF blockade, the pauci-immune pathotype was associated with inadequate response to certolizumab pegol: only 28.6% of patients with pauci-immune synovium achieved clinical response compared with 83.3% of patients with lympho-myeloid or diffuse-myeloid pathotypes (66). R4RA analyses also identified stromal/fibroblast signatures in patients refractory to multiple medications, supporting the concept that fibroblast-dominant synovitis may contribute to multidrug resistance (6).

This pathotype is therefore especially important for the central argument of the review: not all active RA is immunologically equivalent. Some patients may fail biologic therapy not because of insufficient drug exposure, but because the dominant pathological process is stromal, fibroblast-driven, or tissue-remodeling–oriented rather than primarily cytokine- or B-cell–driven. This makes the pauci-immune/fibroblastic pathotype a candidate biomarker of poor response to multiple bDMARDs, while also highlighting the need for therapeutic strategies targeting fibroblast activation, stromal remodeling, and tissue-resident inflammatory niches.

##### 2.2.3.2 Fibroblast subsets as stromal biomarkers of response and resistance

Synovial fibroblasts are no longer viewed as passive structural cells, but as active organizers of inflammation, tissue destruction, and treatment resistance in rheumatoid arthritis. Single-cell and functional studies have demonstrated that fibroblast-like synoviocytes (FLS) are highly heterogeneous and include subsets with distinct anatomical localization, transcriptional programs, and pathogenic functions. This makes fibroblast subsets particularly important for tissue-level biomarker development, especially in patients whose disease is poorly explained by conventional systemic inflammatory markers.

A major conceptual advance was the identification of functionally distinct fibroblast subsets in arthritis. FAPα^+^THY1^+^ fibroblasts, primarily located in the sublining synovium, are associated with inflammatory amplification, whereas FAPα^+^THY1^-^ fibroblasts, enriched in the lining layer, preferentially mediate cartilage and bone damage. Experimental transfer of FAPα^+^THY1^+^ fibroblasts induced more severe and persistent inflammation, while FAPα^+^THY1^-^ fibroblasts selectively promoted bone and cartilage damage with limited inflammatory effects (67). These findings suggest that fibroblast composition may help distinguish patients whose dominant pathology is inflammatory from those in whom tissue-destructive stromal programs predominate.

Single-cell transcriptomic studies further expanded this concept by identifying RA-expanded fibroblast states, including THY1(CD90)^+^HLA-DRA^hi sublining fibroblasts, which may participate in antigen presentation and immune-cell organization within inflamed synovium (68). These data are particularly relevant for biomarker research because they link fibroblast subsets to immune activation rather than only to matrix remodeling. In addition, studies of synovial fibroblast subsets across pathotypes indicate that fibroblast composition differs between lympho-myeloid, diffuse-myeloid, and pauci-immune synovitis, supporting the idea that fibroblast phenotypes are part of broader tissue immunophenotypes rather than isolated stromal features (69). From a therapeutic perspective, fibroblast-rich or pauci-immune synovitis may represent a clinically important state of relative resistance to conventional immune-targeted biologics. In such patients, failure of TNF inhibitors, rituximab, or IL-6 blockade may reflect not only inadequate suppression of inflammation, but also persistence of fibroblast-driven tissue remodeling, matrix invasion, and local survival niches. Thus, fibroblast subsets may serve as biomarkers of stromal-dominant RA, poor response to several bDMARDs, and a future rationale for therapies targeting fibroblast activation, FAPα^+^ subsets, stromal cytokines, or fibroblast–immune cell interactions (68).

##### 2.2.3.3 Macrophage subsets as biomarkers of inflammatory activity, remission, and flare risk

Macrophages are central effector cells in RA synovitis and are among the most informative tissue-resident populations for defining inflammatory state. Unlike blood monocytes or systemic acute-phase reactants, synovial macrophage subsets directly reflect the balance between inflammatory amplification and tissue homeostasis within the joint.

Single-cell transcriptomic profiling of synovial tissue macrophages demonstrated that active RA is enriched for MerTK^-^CD48^+^ inflammatory macrophages, including subsets expressing S100A alarmins, IL1B, and SPP1/osteopontin. These macrophages represent major local sources of pathogenic cytokines such as TNF and IL-6 and can induce chemokine and matrix metalloproteinase production by synovial fibroblasts through contact-dependent mechanisms. In contrast, healthy synovium and sustained remission are characterized by restoration of MerTK^+^ macrophage populations, including TREM2^+^ and LYVE1^+^ subsets, which display immunoregulatory and tissue-repair programs (70).

This macrophage axis has direct biomarker implications. A high proportion of MerTK^+^ synovial tissue macrophages appear to mark tissue-level remission, whereas persistence of MerTK^-^ inflammatory macrophages may indicate residual synovial activity despite clinical improvement. Importantly, the same study suggested that the relative abundance of MerTK^+^ macrophages in remission could predict flare after treatment withdrawal; patients with lower MerTK^+^ proportions were more likely to relapse (70).

Macrophage subsets therefore provide a more refined framework than bulk inflammatory markers such as CRP or ESR. Instead of asking whether inflammation is present, macrophage immunophenotyping may distinguish active inflammatory synovitis, resolving/remission synovium, and flare-prone residual inflammation. This makes macrophage states particularly promising as biomarkers for treatment tapering, remission quality, and monitoring of persistent tissue inflammation.

##### 2.2.3.4 B-cell niches and local humoral immune programs

B-cell biology in RA should be interpreted not only through peripheral autoantibody status, but also through the organization of B-cell niches within synovial tissue. In lympho-myeloid synovitis, B cells, plasma cells, T peripheral helper cells, follicular helper-like cells, and stromal cells form spatially organized microenvironments that support antigen presentation, B-cell survival, local differentiation, and autoantibody production.

CXCL13 is a key organizing chemokine in these niches. It promotes recruitment of CXCR5-expressing B cells and contributes to the organization of B-cell–rich aggregates and tertiary lymphoid structures. Peripheral helper T cells, which accumulate in RA synovium, are an important source of CXCL13 and provide help to B cells in inflamed tissue, thereby linking T-cell activation to local humoral autoimmunity.

This has direct relevance for treatment response. B-cell–rich synovial tissue and humoral gene-expression programs are mechanistically aligned with response to B-cell depletion. In the R4RA biopsy-driven trial, patients with low synovial B-cell molecular signatures had poor response to rituximab and better response to tocilizumab, supporting the concept that local target abundance and pathway dominance can guide biologic selection (6, 65).

However, not all B-cell–rich states are equivalent. Persistence of plasma-cell or plasmablast programs may indicate resistance to rituximab because these differentiated populations are CD20-negative and are not directly depleted by anti-CD20 therapy. Therefore, the clinically useful biomarker may not be “B cells present/absent” but rather the structure of the local humoral niche: naïve/memory B cells, plasma-cell differentiation, CXCL13-driven recruitment, survival signals such as BAFF/APRIL, and BCR clonality. This supports a more refined tissue-immunophenotype model for predicting response to rituximab, abatacept, and potentially future B-cell–directed cellular therapies.

##### 2.2.3.5 Cell–cell interaction networks as biomarkers of pathogenic circuits

A tissue immunophenotype is not fully defined by the abundance of individual cell types. RA synovitis is sustained by communication networks between immune and stromal cells, including macrophage–fibroblast, T-cell–B-cell, fibroblast–endothelial, and plasma-cell–stromal interactions. Therefore, future biomarkers may need to quantify active cell–cell interaction networks rather than isolated cells or molecules.

Computational tools such as CellPhoneDB, CellChat, and NicheNet allow inference of intercellular communication from single-cell or spatial omics data. CellPhoneDB infers communication using curated ligand–receptor complexes, CellChat reconstructs signaling networks from scRNA-seq data, and NicheNet links ligands from sender cells to downstream target-gene programs in receiver cells (71–73).

In RA, biologically relevant interaction axes include CXCL13–CXCR5 for B-cell recruitment, TNF–TNFR and IL-6–IL-6R for cytokine-driven inflammation, BAFF/APRIL–B-cell survival pathways for humoral persistence, and macrophage–fibroblast contact-dependent pathways that induce chemokines and matrix metalloproteinases. Such networks may be more informative than single analytes because they capture the functional relationships that maintain synovitis.

For biomarker development, interaction networks could help define whether a patient’s disease is B-cell niche-dominant, macrophage–fibroblast-dominant, cytokine-dominant, or stromal-remodeling-dominant. This could directly inform therapeutic choice: rituximab for B-cell-driven niches, IL-6 blockade for IL-6-centered networks, TNF inhibition for TNF-driven myeloid–stromal activation, or alternative strategies for fibroblast-dominant disease. However, ligand–receptor inference remains computational and hypothesis-generating unless validated experimentally or spatially. Thus, these networks should be considered emerging biomarkers requiring orthogonal validation.

Recommendations for the use of first-line biologics depending on the synovial tissue/molecular subtype are summarized in **Table 1**.

**Table 1:** Synovial tissue/molecular subtype and first-line biologic recommendation.

Subtype	Biomarker signature	First-line biologic recommendation
Lympho-myeloid	B-cell niches + T-cell interaction	Rituximab (B-cell depletion) or Abatacept (T-cell costimulation block)
Diffuse-myeloid	High macrophage markers, calprotectin, IL-6	IL-6 inhibitor
Pauci-immune	Low immune cell infiltration, fibrotic signature	Consider JAK inhibitors first, but if biologic needed: Abatacept or TNFi
Macrophage subtype	High calprotectin, ESR, CRP	IL-6 inhibitor or TNFi (especially adalimumab, golimumab)
Presence of ectopic lymphoid structures/B-cell niches	RF/ACPA high	Rituximab
Absence of B-cell niches but high T-cell markers	Anti-CarP high	Abatacept
Cell–Cell Interaction Networks	High TNF–IL-1 crosstalk	TNFi
	High IL-6–Th17 axis	IL-6 inhibitor
	High CD80/CD86–CD28 co-stimulation	Abatacept
	High BAFF/APRIL & B-T cell interaction	Rituximab

IL, interleukin; TNF, Tumor necrosis factor; ESR, Erythrocyte Sedimentation Rate; CRP, C-reactive Protein; TNFi, Tumor necrosis factor inhibitors; RF, Rheumatoid Factor; ACPA, Anti-Citrullinated Protein Antibodies; Anti-CarP, Anti-Carbamylated Protein Antibodies; BAFF, B-cell activating factor; APRIL, A proliferation-inducing ligand.

##### 2.2.3.6 scRNA-seq as a platform for defining treatment-relevant cell states

Single-cell RNA sequencing (scRNA-seq) has transformed RA synovial biology by resolving pathogenic cell states that are obscured in bulk tissue profiling. Rather than classifying synovium only by total inflammatory load, scRNA-seq can identify specific populations of fibroblasts, macrophages, T cells, B cells, and monocytes that may be differentially linked to disease activity and therapeutic response.

A landmark integrated analysis combining scRNA-seq, mass cytometry, bulk RNA-seq, and flow cytometry identified 18 synovial cell populations and highlighted RA-expanded states, including THY1(CD90)^+^HLA-DRA^hi sublining fibroblasts, IL1B^+^ pro-inflammatory monocytes, ITGAX^+^TBX21^+^ autoimmune-associated B cells, and PDCD1^+^ peripheral helper T cells. These findings demonstrate that RA synovitis is composed of coordinated pathogenic cell states rather than uniform inflammation (68).

For biomarker development, scRNA-seq has two major advantages. First, it enables identification of cell-type-specific therapeutic targets, such as macrophage-derived IL-1/TNF/IL-6 programs, fibroblast antigen-presentation modules, or CXCL13-producing T-cell states. Second, it allows deconvolution of bulk synovial signatures used in clinical trials such as PEAC and R4RA, helping to determine which exact cell populations drive a predictive molecular signal.

**Figure 3:**
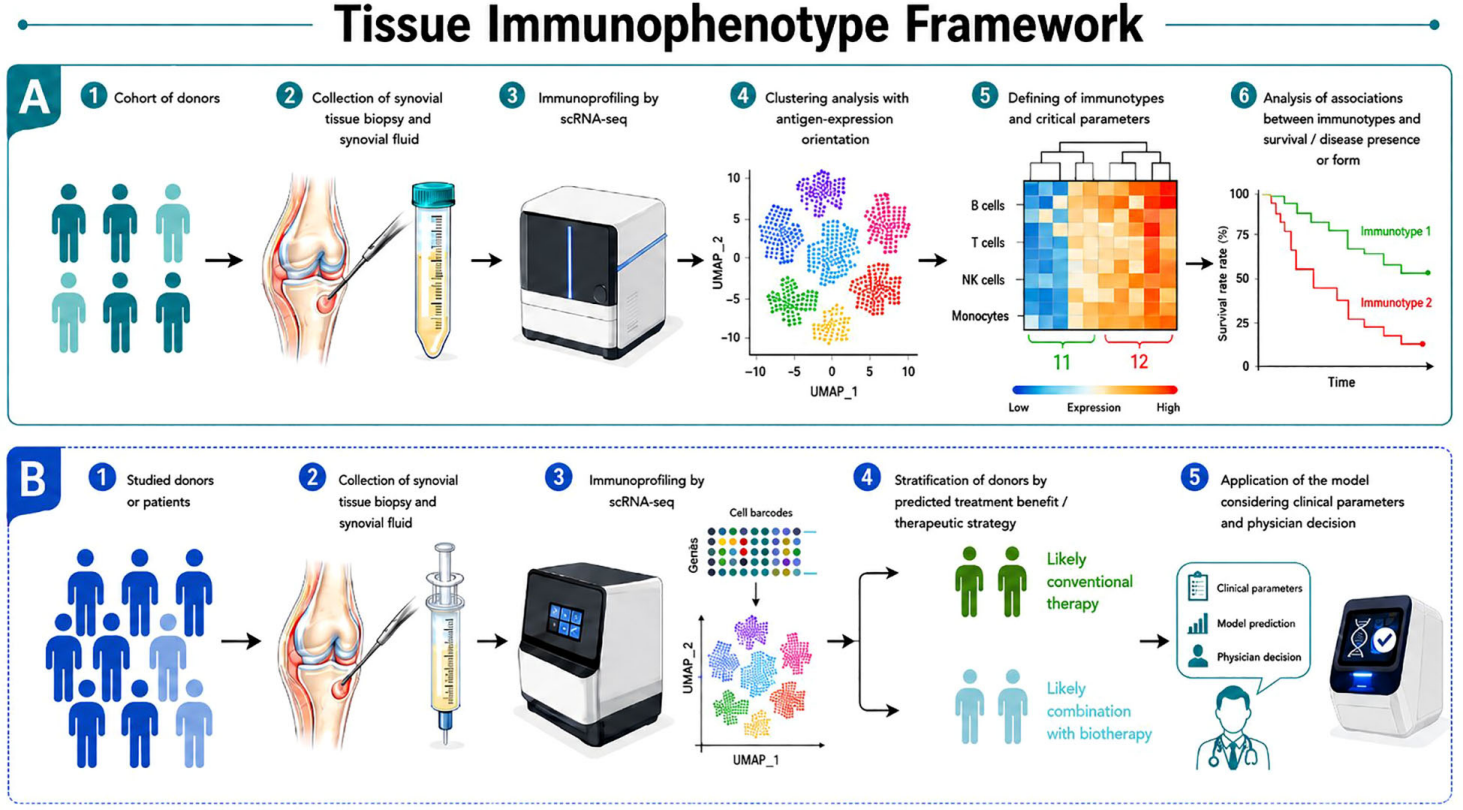
Tissue immunophenotype framework. **(A)** The discovery workflow: donor samples are collected and immunoprofiled using FC analysis, scRNA-seq, or bulk RNA-seq. The resulting data are clustered to define immunotypes and key immune parameters, which are then associated with disease status, disease form, or survival. **(B)** The translational workflow: patient samples are analyzed by flow cytometry, assigned to predefined immunotypes, and stratified according to disease risk, likely disease status, or expected therapeutic benefit. Additional testing may then be used to refine diagnosis or treatment selection.

At present, scRNA-seq is not yet a scalable routine clinical assay. Its value is primarily in discovery and in defining simplified biomarker panels that can later be translated into targeted PCR, flow cytometry, immunohistochemistry, or multiplex imaging assays. Thus, scRNA-seq should be presented in the manuscript not as an immediate clinical test, but as the key technology that enables the shift from descriptive biomarkers to mechanism-defined tissue immunophenotype. (**Figure 3**).

### 2.3 Biomarkers of toxicity

Toxicity biomarkers aim to identify patients at higher risk of adverse events or to detect toxicity early enough to prevent serious harm.

#### 2.3.1 Pharmacogenetic markers

MTX toxicity (hepatic, hematologic, mucosal) has long motivated pharmacogenetic research. A meta-analysis evaluated MTHFR polymorphisms in relation to MTX efficacy and toxicity, reflecting early evidence that variants in folate metabolism may influence adverse-event risk, although effect sizes and clinical utility vary (74).

Beyond MTHFR, variants such as RFC1 have been meta-analyzed for association with MTX efficacy and toxicity in RA, illustrating the broader folate/transport pathway relevance and persistent challenges in reproducibility and implementation (75).

#### 2.3.2 Immunogenicity

While ADA are typically framed as efficacy biomarkers (loss of response), they also relate to adverse reactions (e.g., infusion reactions with some agents) and can inform switching strategies. For example, for tocilizumab, immunogenicity has been examined in RA, including studies assessing ADA and drug levels; larger analyses indicate generally low immunogenicity risk across routes and regimens (76).

## 3 Drug specific biomarkers

Given the central role of effect of several biomarkers in the treatment algorithm of RA, this review focuses on biomarkers associated with response to the most commonly used agents in routine clinical practice. Although multiple therapeutic options exist there are several therapies which are considered as the most effective in RA.

In this section, we summarize current evidence on clinical, pharmacological, genetic, molecular, and imaging biomarkers that have been investigated in relation to treatment response to these therapies. Emphasis is placed on biomarkers with potential relevance for treatment stratification, early identification of inadequate responders, dose optimization, and prediction of adverse events. By structuring the discussion according to individual therapies, we aim to highlight both mechanism-aligned biomarker strategies and shared methodological challenges in translating candidate predictors into clinically actionable tools. (**Table 2**).

**Table 2:** List of known drug-specific biomarkers.

Drug	Biomarker	How the biomarker modifies therapeutic response	Trial characteristics	Reference
Methotrexate	RBC Methotrexate polyglutamates (MTX-PGs)	Higher intracellular MTX-PG levels are associated with improved clinical response; may also correlate with toxicity risk	Retrospective study; 395 patients,401 MTX-PG concentrations and 1,337 DAS28 measurements were available between 0 and 300 days after MTX treatment onset	(60)
Methotrexate	MTHFR polymorphisms	Variants influence MTX metabolism; associated with variability in response and toxicity	Meta-analysis	(74)
Methotrexate	RFC1 (SLC19A1) polymorphisms	Affect MTX transport and intracellular accumulation; linked to response heterogeneity	Meta-analysis	(75)
Leflunomide	Teriflunomide plasma levels	Higher and stable exposure associated with better disease control	Retrospective study, 67 patients	(80)
Leflunomide	DHODH polymorphisms	Variants in drug target gene associated with differential clinical response	–	(81)
Hydroxychloroquine	Whole-blood HCQ concentration	Higher levels weakly correlate with improved response; excessive levels linked to toxicity	Retrospective study; 123 available patients	(88)
TNF inhibitors	Rheumatoid factor (RF) levels	Higher rheumatoid factor (RF) levels connected to lower TNFi drug levels and higher risk of secondary non-response	Retrospective study; 170 available patients	(92)
TNF inhibitors	CXCL10/CXCL13	Higher baseline levels associated with better TNFi response in selected cohorts	Retrospective study; 29 available patients	(94)
TNF inhibitors	Calprotectin (baseline)	Elevated levels may predict response to TNF blockade	Retrospective study; 170 available patients	(118)
Rituximab	Synovial B-cell gene signature	B-cell–rich synovium predicts better response to anti-CD20 therapy	Retrospective study; 164 available patients	(65)
Rituximab	Plasmablast gene signature	Associated with non-response due to CD20-negative cell populations	Retrospective study; patients from REFLEX, DANCER, SCRIPT, SERENE trials	(96)
Rituximab	BCR repertoire dominance (early post-treatment)	Persistence of dominant baseline clones predicts clinical non-response	Retrospective study; 14 patients	(97)
Rituximab	Early naïve BCR repopulation	Associated with later improvement in disease activity	Retrospective study; 28 patients	(98)
Tocilizumab	IL6R polymorphisms	Certain variants associated with differential treatment response	Retrospective study; 591 patients	(100)
Tocilizumab	IL6R polymorphisms	IL-6 receptor gene polymorphism rs4845625 was associated with the efficacy of response to tocilizumab therapy at week 6 of therapy	Retrospective study, 88 patients	(119)
Tocilizumab	GM-CSF	High GM-CSF levels at baseline are a marker of high disease activity and a predictor of poor response to TCZ	Prospective cohort study, 174 patients	(120)
Abatacept	ACPA status	ACPA-positive RA shows better response to costimulation blockade	Retrospective study; 646 patients	(39)
Abatacept	Type I IFN gene signature	IFN-related transcriptomic profile associated with improved response	Retrospective study; 45 patients	(8)
Abatacept	CTLA4 polymorphisms	Genetic variants in costimulation pathway linked to better EULAR response	Retrospective study; 109 patients	(101)
JAK inhibitors	Baseline transcriptomic signatures	Certain immune gene-expression patterns associated with response (exploratory)	Retrospective study; 28 patients	(102)
JAK inhibitors	Composite multi-omic models	Machine-learning–derived panels may predict response but require validation	Retrospective study; 264 patients	(104)

### 3.1 Biomarkers of response to conventional synthetic DMARDs

Conventional synthetic DMARDs remain the cornerstone of rheumatoid arthritis therapy, both as first-line treatment and as supporting.

therapy in combination with biologic or targeted synthetic agents. Despite their established efficacy, clinical responses to DMARDs vary substantially among patients, reflecting the marked heterogeneity of RA pathogenesis, differences in pharmacokinetics and pharmacodynamics, genetic determinants of drug metabolism, and variability in inflammatory and synovial tissue phenotypes.

Importantly, accumulating evidence suggests that integrated biomarker models are likely to be more informative than single-marker approaches, although robust clinical implementation remains limited (77).

#### 3.1.1 Methotrexate

MTX is widely regarded as the anchor conventional synthetic DMARD in rheumatoid arthritis. Its anti-inflammatory effects are mediated through inhibition of folate-dependent enzymes involved in purine metabolism and through increased extracellular adenosine levels, which suppress multiple inflammatory pathways. Among all csDMARDs, MTX has the most extensively studied biomarker landscape, reflecting both its central role in therapy and the substantial variability in treatment response.

One of the most extensively investigated pharmacological biomarkers is the measurement of erythrocyte methotrexate polyglutamates (RBC MTX-PGs). Upon entering the cells, MTX is converted into polyglutamated derivatives (MTX-PG1–PG5), which accumulate intracellularly and prolong the effect of the drug. Since red blood cells gradually accumulate MTX-PG during their lifetime, RBC MTX-PG levels serve as a surrogate marker of the cumulative intracellular effect of the drug (60). Thus, the biomarker directly reflects the effect of MTX at the cellular level, indicating its absorption and metabolism and helping to select the necessary dosage for each patient.

However, several limitations restrict its clinical implementation. First, studies consistently demonstrate substantial interpatient variability in MTX-PG accumulation despite similar dosing regimens. This variability is influenced by multiple factors, including genetic polymorphisms affecting folate metabolism, differences in drug transport across cell membranes, renal function, route of administration (oral vs subcutaneous), and patient characteristics such as age or body mass index. Second, RBCs are not the primary pharmacological target of MTX, and therefore erythrocyte MTX-PG levels represent only an indirect proxy for drug exposure in immune cells within synovial tissue. Third, because erythrocytes have a long lifespan (~120 days), MTX-PG levels change slowly and therefore may not be optimal for rapid therapeutic decision-making.

Importantly, MTX-PG concentrations may also correlate with toxicity risk, including hepatic and hematologic adverse events (78). This dual relationship suggests that MTX-PG levels could potentially function as both efficacy and safety biomarkers, enabling identification of patients at risk for underexposure or excessive accumulation. Nevertheless, clinical thresholds remain poorly standardized, and prospective trials evaluating MTX-PG–guided dosing strategies are still limited.

Another major area of biomarker research involves pharmacogenetic predictors of MTX response. Genetic polymorphisms in enzymes and transporters involved in folate metabolism and MTX pharmacokinetics-such as MTHFR, ATIC, and RFC1 (SLC19A1)-have been studied extensively. Meta-analyses have reported associations between certain polymorphisms and variability in both treatment response and toxicity risk (74). For example, variants in *MTHFR* may influence folate metabolism and intracellular MTX effects, while polymorphisms in *SLC19A1* affect MTX transport into cells. The main advantage of pharmacogenetic biomarkers is that they can be assessed prior to treatment initiation, potentially enabling pre-treatment stratification of patients. However, the clinical value of these markers remains limited because effect sizes are modest and results vary across ethnic populations and study designs, reducing reproducibility and preventing routine clinical use.

More recently, multiomics approaches, including metabolomics and proteomics, have been explored to identify broader molecular signatures associated with MTX response. Systematic reviews have identified a growing number of metabolomic and proteomic candidate biomarkers potentially associated with MTX treatment response in rheumatoid arthritis. These include alterations in purine and pyrimidine metabolism intermediates, amino acid pathways, lipid mediators, and inflammatory protein networks, reflecting the multifaceted pharmacodynamic effects of MTX on immune activation and cellular metabolism. Because MTX exerts pleiotropic effects-ranging from modulation of folate-dependent pathways to regulation of adenosine-mediated anti-inflammatory signaling-multiomics biomarkers have been proposed as a way to capture global biological responses to therapy rather than isolated molecular events (79). In principle, such integrative biomarkers could provide a more comprehensive picture of treatment response by reflecting multiple biological processes simultaneously. However, the main challenge in this field remains poor reproducibility between cohorts and lack of external validation, as many identified signatures are derived from relatively small datasets and use heterogeneous analytical platforms.

Overall, MTX biomarker research illustrates both the promise and the challenges of precision medicine in rheumatoid arthritis. Pharmacological markers such as MTX-PGs currently represent the most biologically plausible indicators of drug exposure, while pharmacogenetic markers offer the possibility of pre-treatment stratification. However, neither approach alone has achieved sufficient predictive accuracy for routine clinical implementation. Future strategies will likely require integrative models combining pharmacokinetic, genetic, and immunological biomarkers to better predict treatment response and guide individualized MTX therapy.

Current evidence suggests that MTX polyglutamates represent one of the most biologically plausible pharmacological biomarkers in RA because they directly reflect intracellular drug exposure. However, their clinical utility remains limited. Although multiple studies demonstrate associations between higher MTX-PG concentrations and improved clinical response, the reported effect sizes are modest and substantial interindividual variability persists despite similar dosing regimens. Furthermore, erythrocyte MTX-PGs represent a surrogate rather than a direct measurement of drug activity in synovial immune cells. Most importantly, no universally accepted therapeutic thresholds currently exist, and prospective MTX-PG-guided treatment strategies have not consistently demonstrated superiority over standard treat-to-target approaches. Consequently, MTX-PGs are best viewed as mechanistically informative biomarkers rather than clinically validated tools for routine treatment selection.

#### 3.1.2 Leflunomide

Leflunomide (LEF) is a conventional synthetic DMARD whose immunomodulatory activity is mediated by its active metabolite teriflunomide. Teriflunomide inhibits dihydroorotate dehydrogenase (DHODH), a mitochondrial enzyme required for *de novo* pyrimidine synthesis, thereby suppressing proliferation of activated lymphocytes, particularly T and B cells. Because the drug mechanism is relatively well defined, biomarker research for leflunomide has largely focused on pharmacokinetic variability in teriflunomide exposure and pharmacogenetic variation in the DHODH pathway, providing a mechanistically grounded framework for biomarker development.

One of the most direct pharmacological biomarkers investigated for LEF therapy is the plasma concentration of teriflunomide, which reflects systemic drug exposure. Precision medicine approaches suggest that variability in teriflunomide levels may partially explain differences in treatment response between patients receiving similar dosing regimens. Pharmacokinetic studies have shown that patients with higher steady-state concentrations of teriflunomide are more likely to achieve improvements in disease activity, supporting the concept that drug exposure itself can serve as a biomarker of therapeutic response (80). The main advantage of this biomarker is its direct mechanistic relevance: because teriflunomide is the active compound responsible for DHODH inhibition, its circulating concentration provides a biologically meaningful measure of pharmacological activity.

However, several limitations affect the clinical applicability of teriflunomide level monitoring. Teriflunomide has an extremely long half-life (approximately two weeks) due to extensive enterohepatic recirculation, which leads to relatively stable plasma concentrations over time. As a result, interindividual variability in exposure may be less pronounced than with some other DMARDs, limiting the dynamic range of this biomarker. In addition, standardized therapeutic thresholds for optimal drug concentrations have not been clearly established, and routine therapeutic drug monitoring for leflunomide is not currently recommended in clinical practice.

In parallel, pharmacogenetic biomarkers targeting the drug’s molecular pathway have been investigated. Since DHODH is the direct target of teriflunomide, genetic polymorphisms within the DHODH gene represent biologically plausible candidates for predicting treatment response. Studies have reported associations between specific DHODH variants, including rs3213422, and improved likelihood of achieving controlled disease activity during leflunomide therapy (81). These findings suggest that genetic variation affecting enzyme structure or expression may influence sensitivity to DHODH inhibition and therefore treatment efficacy. The main strength of such biomarkers lies in their mechanism-based rationale and the possibility of performing genetic testing before treatment initiation, enabling potential pre-treatment stratification of patients.

Nevertheless, pharmacogenetic associations for DHODH remain preliminary. Most available studies involve relatively small cohorts, and the reported effect sizes are moderate. Furthermore, replication across independent populations has been limited, and it remains unclear whether these variants have sufficient predictive value to guide clinical decision-making.

In addition to biomarkers of efficacy, several studies have investigated genetic predictors of leflunomide toxicity and treatment discontinuation. Polymorphisms in genes involved in drug metabolism, particularly CYP1A2 and CYP2C19, have been associated with variability in leflunomide metabolism and tolerability (82). Variants in these enzymes may alter the metabolic clearance of teriflunomide and contribute to differences in drug accumulation, potentially increasing the risk of adverse events such as hepatotoxicity or gastrointestinal intolerance. These pharmacogenetic markers therefore represent potential toxicity-oriented biomarkers, which could help identify patients at increased risk of adverse reactions.

Overall, biomarker research for leflunomide illustrates one of the clearest examples in RA pharmacology where biomarker development aligns closely with drug mechanism. Pharmacokinetic markers such as teriflunomide exposure primarily relate to treatment efficacy, while pharmacogenetic markers targeting DHODH may influence drug sensitivity, and metabolic enzyme variants may help predict toxicity risk. Despite this strong biological rationale, current evidence remains insufficient for routine clinical implementation due to limited cohort sizes, lack of standardized thresholds, and insufficient prospective validation (83). Future studies integrating pharmacokinetic, pharmacogenetic, and immunological biomarkers may improve the predictive accuracy of leflunomide response models and facilitate more personalized therapeutic strategies.

#### 3.1.3 Sulfasalazine

Sulfasalazine (SSZ) is a conventional synthetic DMARD with anti-inflammatory properties that are thought to involve inhibition of NF-κB signaling, suppression of pro-inflammatory cytokine production, and modulation of immune cell activation. However, the precise mechanism of action of sulfasalazine in rheumatoid arthritis remains incompletely defined. After oral administration, SSZ is cleaved by intestinal bacteria into two main metabolites: 5-aminosalicylic acid and sulfapyridine, the latter being considered the primary pharmacologically active moiety in RA. This metabolic pathway has strongly influenced biomarker research for SSZ, which has largely focused on drug metabolism and acetylation phenotypes rather than direct immunological markers.

Early pharmacokinetic studies investigated whether serum concentrations of sulfasalazine or its metabolites could predict clinical response. Some reports suggested that treatment efficacy might be dose-dependent, raising the possibility that higher systemic exposure could correlate with improved therapeutic outcomes. However, subsequent studies demonstrated that measured concentrations of SSZ and its metabolites showed inconsistent associations with clinical efficacy, and no reliable exposure–response relationship was established (84). These findings significantly limited the potential utility of therapeutic drug monitoring for predicting treatment response in SSZ-treated patients.

Because sulfapyridine undergoes hepatic metabolism through N-acetylation, attention subsequently shifted to pharmacogenetic biomarkers related to acetylation capacity. Individuals can be broadly classified as slow or fast acetylators, primarily determined by genetic variation in the N-acetyltransferase 2 (NAT2) enzyme. Early clinical studies suggested that acetylator status might influence treatment response, with slow acetylators potentially achieving higher systemic exposure to sulfapyridine. However, the relationship between acetylator phenotype and therapeutic efficacy proved inconsistent across studies and appeared to depend heavily on study design, patient populations, and clinical endpoints (85). As a result, NAT2 status has not been established as a reliable predictor of SSZ efficacy.

In contrast, much stronger evidence supports the role of NAT2 polymorphisms as predictors of sulfasalazine toxicity. Because slow acetylators metabolize sulfapyridine more slowly, they tend to accumulate higher concentrations of this metabolite, which may increase the risk of adverse drug reactions such as gastrointestinal intolerance, hepatotoxicity, and hematologic abnormalities. A systematic review and meta-analysis demonstrated that the NAT2 slow-acetylator genotype is significantly associated with an increased risk of adverse reactions to sulfasalazine, making it one of the most robust pharmacogenetic biomarkers of drug tolerability identified for csDMARD therapy (86). From a mechanistic perspective, this association is biologically plausible, as the accumulation of sulfapyridine—the metabolite primarily responsible for systemic effects in RA—directly depends on acetylation capacity (87).

Overall, biomarker development for sulfasalazine differs from that of other DMARDs in that the most clinically relevant markers relate primarily to drug safety rather than treatment efficacy. While pharmacokinetic biomarkers such as serum drug levels and pharmacogenetic markers such as NAT2 acetylator status have been explored, only the latter has demonstrated relatively consistent predictive value, specifically for toxicity risk. However, even NAT2 testing has not yet been widely incorporated into routine clinical decision-making, partly due to variability in reported effect sizes and the declining use of sulfasalazine as monotherapy in modern RA treatment algorithms.

#### 3.1.4 Hydroxychloroquine

Hydroxychloroquine (HCQ) is a conventional synthetic DMARD that exerts immunomodulatory effects through interference with lysosomal activity, endosomal acidification, and Toll-like receptor (TLR) signaling, particularly TLR7 and TLR9. These mechanisms reduce antigen presentation, inhibit activation of dendritic cells, and attenuate downstream inflammatory responses, including cytokine production and B-cell activation. Although HCQ is generally less potent than methotrexate or leflunomide in controlling synovial inflammation, it remains widely used in combination treatment regimens and in patients with milder disease phenotypes due to its favorable safety profile.

Biomarker research for hydroxychloroquine has primarily focused on pharmacokinetic markers, particularly the relationship between circulating HCQ concentrations and therapeutic response. HCQ has a complex pharmacokinetic profile characterized by extensive tissue distribution, intracellular accumulation, and a very long elimination half-life. Because of this, whole-blood HCQ levels can serve as an indicator of cumulative drug exposure. Early pharmacokinetic studies demonstrated that higher concentrations of HCQ and its metabolites were weakly associated with improved clinical outcomes, suggesting a possible exposure–response relationship (88). However, these associations were relatively modest, indicating that HCQ levels alone cannot reliably predict treatment success.

Despite the relatively weak correlation with efficacy, measurement of HCQ concentrations may still have practical clinical utility in certain contexts. In particular, therapeutic drug monitoring can help identify poor adherence, which is a recognized issue in chronic rheumatic diseases. Patients with unexpectedly low HCQ concentrations despite prescribed treatment may be experiencing suboptimal therapeutic exposure due to inconsistent medication use. Conversely, excessively high concentrations have been associated with an increased risk of adverse effects, including gastrointestinal intolerance and, potentially, retinal toxicity. As a result, HCQ level monitoring has been proposed as a tool for adherence assessment and safety monitoring, although standardized therapeutic ranges have not been firmly established for RA (89).

In addition to pharmacokinetic biomarkers, several studies have investigated immunological and pharmacodynamic markers associated with HCQ treatment. For example, HCQ has been shown to influence cytokine networks and B-cell–related signaling pathways, including modulation of B-cell activating factor (BAFF) levels. Because BAFF plays an important role in B-cell survival and autoantibody production, changes in BAFF concentrations during HCQ therapy have been explored as potential indicators of drug activity (90). These observations support the biological plausibility that HCQ may exert immunomodulatory effects through regulation of B-cell–mediated immune responses.

However, these immunological markers remain largely exploratory and have not yet been validated as predictive biomarkers of treatment response. Most studies examining cytokine or BAFF modulation involve relatively small patient cohorts, and the relationship between these pharmacodynamic changes and clinical outcomes remains uncertain. Furthermore, because HCQ acts through multiple cellular pathways and has relatively modest anti-inflammatory potency compared with other DMARDs, identifying a single robust biomarker of response has proven challenging.

Overall, biomarker development for hydroxychloroquine remains relatively limited compared with other csDMARDs. Pharmacokinetic monitoring of HCQ levels may provide useful information regarding drug exposure and adherence, while immunological markers such as BAFF modulation offer insight into potential mechanisms of action. However, neither approach has yet produced biomarkers with sufficient predictive accuracy to guide routine therapeutic decision-making in rheumatoid arthritis.

### 3.2 Biomarkers of response to biologic DMARDs

Biologic DMARDs and targeted synthetic DMARDs (including JAK inhibitors) have transformed RA outcomes by directly interrupting key immune pathways (e.g., TNF, IL-6, B cells, T-cell costimulation, JAK/STAT). Yet primary non-response and secondary loss of response remain common, reflecting RA’s molecular heterogeneity and the fact that different patients can have distinct “dominant” inflammatory circuits. Systematic reviews emphasize that many candidate markers show promise results but often fail external validation; the most actionable biomarkers today are typically those related to immunogenicity and exposure (especially for monoclonal antibodies) and tissue molecular signatures in biopsy-driven studies (91).

#### 3.2.1 TNF inhibitors

Tumor necrosis factor inhibitors (TNFi) exert their therapeutic effects by neutralizing tumor necrosis factor-α (TNF-α), a key pro-inflammatory cytokine driving synovial inflammation, immune cell activation, and joint destruction in rheumatoid arthritis. By blocking TNF signaling, these agents suppress downstream inflammatory cascades, including activation of NF-κB pathways, cytokine production, leukocyte recruitment, and synovial angiogenesis.

Among biomarkers of TNF inhibitor response, the most clinically actionable are pharmacological and immunogenicity-related markers, particularly through drug concentrations and anti-drug antibodies (ADA). These biomarkers are used within the framework of therapeutic drug monitoring (TDM) and can help distinguish between pharmacokinetic failure (insufficient drug exposure due to rapid clearance or immunogenicity) and pharmacodynamic failure, in which adequate drug levels are present but the underlying inflammatory pathway is not TNF-driven. In clinical practice, low trough drug levels or the presence of ADA may support strategies such as dose escalation or switching within the TNFi class, whereas adequate drug levels in the absence of response often favor switching to a therapy with a different mechanism of action.

Recent real-world data further suggest that patient immunological characteristics may influence TNFi pharmacokinetics. A large observational study demonstrated that RF positivity was associated with lower circulating TNFi concentrations and an increased risk of secondary loss of response, suggesting that autoantibody status may affect drug exposure and long-term treatment durability (92). These findings highlight the potential interaction between humoral autoimmunity and biologic drug pharmacokinetics.

Beyond pharmacological monitoring, numerous circulating inflammatory mediators have been investigated as potential predictors of TNFi response. However, a systematic review evaluating a wide range of candidate biomarkers concluded that few markers demonstrate consistent predictive accuracy across independent cohorts, largely due to heterogeneity in study design, patient populations, and outcome measures (93). Nevertheless, several immune mediators have shown relatively reproducible associations. For example, chemokines such as CXCL10 and CXCL13 have been associated with improved clinical response to TNF inhibitors in prospective RA cohorts. These molecules reflect activation of specific immune pathways, including lymphocyte recruitment and B-cell–associated inflammation, and may indicate disease states in which TNF-driven inflammatory networks are particularly dominant (94).

More recently, precision-medicine approaches have begun to integrate cytokine and chemokine panels with molecular and synovial tissue features to stratify patients according to their likelihood of responding to TNF blockade. Such approaches attempt to identify composite inflammatory signatures reflecting TNF-dependent disease biology. Early studies suggest that combining multiple biomarkers may improve predictive accuracy compared with single markers, although assay standardization and prospective validation remain major challenges for clinical implementation (95).

Overall, biomarker research for TNF inhibitors illustrates the diversity of approaches currently explored in RA precision medicine. Pharmacological biomarkers, such as drug levels and ADA, remain the most clinically applicable tools for guiding treatment adjustments, while immune mediators and multi-marker signatures offer promising insights into the biological mechanisms underlying therapeutic response. However, most candidate biomarkers require further validation in large prospective studies before they can be routinely implemented in clinical decision-making.

#### 3.2.2 Rituximab (anti-CD20 therapy)

Rituximab is a monoclonal antibody targeting CD20, leading to depletion of circulating and tissue CD20+ B lymphocytes. Through this mechanism, rituximab reduces autoantibody production, disrupts antigen presentation by B cells, and attenuates B-cell–mediated immune activation in rheumatoid arthritis.

Among clinical biomarkers, the most robust and widely used predictors of rituximab response are serological autoantibodies, particularly rheumatoid factor (RF) and anti-citrullinated protein antibodies (ACPA). Seropositive patients generally demonstrate a higher likelihood of clinical response to B-cell depletion therapy, which is thought to reflect a stronger contribution of B-cell–driven autoimmunity to disease pathogenesis in this subgroup. As a result, serological status remains one of the few biomarkers routinely considered in clinical decision-making for rituximab therapy.

Beyond serological markers, recent research has increasingly focused on synovial tissue biomarkers, reflecting the concept that therapeutic response may depend on the presence of the drug target within the inflamed tissue. Biopsy-driven studies, including the R4RA trial, have demonstrated that synovial B-cell–rich molecular signatures, including humoral immune gene expression programs, are associated with differential treatment responses. Patients with strong B-cell–related transcriptional signatures in the synovium were more likely to benefit from rituximab, supporting the principle that target presence and pathway dominance within synovial tissue are key determinants of therapeutic response (65).

Additional mechanistic studies have highlighted the importance of B-cell differentiation states in determining rituximab responsiveness. In particular, a study published in *Science Translational Medicine* identified a plasmablast-associated transcriptional signature that was associated with poor clinical response to anti-CD20 therapy (96). Because plasmablasts and plasma cells do not express CD20, their expansion may allow pathogenic B-cell responses to persist despite rituximab-mediated depletion of earlier B-cell populations.

More recently, advances in adaptive immune receptor repertoire sequencing (AIRR-seq) have enabled detailed characterization of B-cell receptor (BCR) repertoires during rituximab therapy. These studies suggest that treatment outcomes may depend not only on the extent of B-cell depletion but also on the clonal architecture of the B-cell compartment. For example, a study Pollastro S. et al. (97) demonstrated that peripheral blood BCR repertoires four weeks after rituximab treatment contained fewer but more somatically mutated clones, while changes in synovial BCR repertoires occurred more gradually. Importantly, patients who failed to respond to therapy exhibited greater persistence of dominant baseline BCR clones and higher clonal overlap with the pre-treatment repertoire, suggesting that incomplete disruption of pathogenic B-cell clones may underlie treatment resistance.

Subsequent work further explored the dynamics of B-cell depletion and repopulation using repertoire-based metrics. In a prospective study (98), investigators used the proportion of unmutated BCR sequences as a marker of naïve B-cell repopulation following rituximab therapy. Interestingly, early B-cell depletion within the first month after treatment did not reliably predict long-term clinical outcomes. In contrast, early repopulation with unmutated BCR sequences within six months was associated with a significant reduction in disease activity between months six and twelve. These findings suggest that qualitative features of B-cell reconstitution—potentially reflecting restoration of a more naïve, non-autoreactive B-cell repertoire-may be linked to improved clinical outcomes. The study also observed a non-significant trend suggesting that anti-drug antibodies might influence the kinetics of B-cell repopulation.

Taken together, rituximab provides one of the clearest examples in rheumatoid arthritis of how mechanism-aligned biomarkers can inform therapeutic response. Evidence suggests that B-cell–rich synovial tissue states and humoral immune signatures tend to predict favorable responses to B-cell depletion, whereas disease states dominated by plasmablast or plasma-cell biology may be relatively resistant to anti-CD20 therapy. Nevertheless, widespread clinical implementation of these biomarkers remains limited by the need for standardized assays, simplified diagnostic platforms, and prospective validation in large patient cohorts.

#### 3.2.3 IL-6 pathway inhibitors (tocilizumab)

Tocilizumab is a monoclonal antibody targeting the interleukin-6 receptor (IL-6R), thereby inhibiting IL-6–mediated signaling pathways that drive systemic inflammation, synovial activation, and acute-phase responses in rheumatoid arthritis. By blocking both membrane-bound and soluble IL-6 receptors, tocilizumab suppresses downstream JAK–STAT signaling, leading to reduced cytokine production, decreased leukocyte recruitment, and inhibition of inflammatory cascades within the synovium.

Biomarker research for IL-6 receptor blockade has primarily focused on components of the IL-6 signaling pathway, including circulating cytokines, acute-phase proteins, and genetic variants affecting IL-6 signaling. However, despite the strong mechanistic rationale, clinically validated predictive biomarkers remain limited. A comprehensive review of candidate biomarkers for IL-6 receptor inhibitors concluded that many proposed markers show inconsistent results across studies, largely due to differences in study design, patient populations, and outcome definitions (99). Nevertheless, some of the more reproducible signals have been observed among serum inflammatory proteins and pharmacological exposure-related markers, suggesting that treatment response may partly depend on both baseline inflammatory activity and drug pharmacokinetics.

Genetic biomarkers have also been investigated as potential predictors of response to tocilizumab. Several studies have explored polymorphisms within the IL6 and IL6R genes, as well as variants in downstream signaling pathways. A meta-analysis examining genetic predictors of biologic therapy response reported that certain polymorphisms within the IL-6 signaling axis may be associated with differential clinical outcomes during tocilizumab treatment (100). However, the predictive value of these variants remains modest, and the results are not consistently replicated across independent cohorts, limiting their applicability in routine clinical practice.

A particular challenge in evaluating biomarkers of response to tocilizumab arises from the unique biology of IL-6 signaling and its effects on acute-phase markers. IL-6 is a key regulator of hepatic production of CRP and other acute-phase reactants. Consequently, IL-6 receptor blockade leads to rapid and profound suppression of CRP levels, often within days of treatment initiation. While this pharmacodynamic effect confirms effective pathway inhibition, it complicates the interpretation of CRP as a biomarker of treatment response. In many cases, CRP reduction reflects direct pharmacological blockade of IL-6 signaling rather than true resolution of underlying synovial inflammation, which may lead to discordance between laboratory markers and clinical disease activity (99).

Overall, biomarker development for tocilizumab highlights the challenges of translating mechanism-based biological insights into clinically useful predictive tools. Although candidate biomarkers within the IL-6 pathway—including cytokine levels, genetic polymorphisms, and pharmacokinetic markers—have been identified, none have yet demonstrated sufficient predictive accuracy for routine use. Future biomarker strategies may therefore require integrative approaches combining molecular, genetic, and synovial tissue signatures to better identify patients whose disease is predominantly driven by IL-6–dependent inflammatory pathways.

#### 3.2.4 Abatacept

Abatacept is a CTLA-4–Ig fusion protein that inhibits T-cell activation by blocking the CD80/CD86–CD28 costimulatory pathway, a critical signal required for full activation of naïve T cells. By interfering with this costimulatory interaction, abatacept suppresses downstream T-cell–mediated immune responses, including cytokine production, B-cell help, and autoantibody generation, thereby reducing synovial inflammation in rheumatoid arthritis.

Clinical and immunological heterogeneity of RA appears to influence response to costimulation blockade. In particular, studies have demonstrated that ACPA-negative RA patients often show lower response rates to abatacept compared with ACPA-positive patients, suggesting that the adaptive immune system may play a less dominant pathogenic role in this RA subtype (39). Because abatacept directly targets T-cell–dependent immune activation, therapies that interfere with this pathway may be more effective in disease states characterized by stronger adaptive immune involvement, such as seropositive RA.

Consequently, biomarker research for abatacept has largely focused on molecular signatures reflecting T-cell activation and immune signaling pathways, particularly interferon-related transcriptional programs. Transcriptomic studies have shown that type I interferon (IFN) gene signatures may be associated with improved response to abatacept therapy (8). These findings suggest that specific immune activation states, characterized by interferon-driven inflammatory pathways, may identify patient subgroups whose disease is more dependent on adaptive immune responses and therefore more amenable to costimulation blockade.

In addition to transcriptomic biomarkers, several studies have investigated pharmacogenetic predictors of abatacept response. Genetic variants within genes involved in T-cell costimulation pathways, including CTLA4 polymorphisms, have been associated with improved clinical response and higher rates of achieving low disease activity or EULAR response in abatacept-treated patients (101). These findings support the biological plausibility that host genetic variation within the drug’s target pathway may influence therapeutic outcomes. However, as with many pharmacogenetic biomarkers in RA, the reported associations require further validation in larger and ethnically diverse cohorts before they can be implemented in clinical practice.

Beyond classical serological markers such as RF and ACPA, additional autoantibody profiles have also been explored as potential predictors of abatacept response. For example, anti-carbamylated protein antibodies (anti-CarP) have been investigated as candidate biomarkers reflecting alternative pathways of autoimmunity. Some studies suggest that the presence of anti-CarP antibodies may correlate with treatment outcomes during abatacept therapy, although the available evidence remains limited and heterogeneous across patient populations (47).

Overall, biomarker research for abatacept highlights the importance of immune pathway–specific predictors in RA precision medicine. Current evidence suggests that molecular signatures reflecting T-cell activation and interferon-related immune states, along with selected pharmacogenetic markers within the costimulation pathway, represent the most promising candidates for predicting response to abatacept. However, these biomarkers remain largely investigational, and further prospective studies are needed to establish their clinical utility and reproducibility across different patient cohorts.

The selection of biological agents based on biomarker types in RA is shown in **Table 3**.

**Table 3:** Biologic drug selection in RA based on biomarkers types.

Biologic drugs	Indicated by biomarker profile
TNF inhibitors	High ESR, CRP, diffuse-myeloid, TNF network dominant, no B-cell niches
IL-6 inhibitors	High ESR, CRP, calprotectin, 14-3-3η, macrophage subtype, diffuse-myeloid, pauci-immune with high IL-6
Abatacept	High anti-CarP, lympho-myeloid type, high T-cell co-stimulation markers, absence of B-cell niches
Rituximab	B-cell niches present, RF/ACPA/anti-CarP high, lympho-myeloid with ectopic germinal centers

TNF, Tumor necrosis factor; ESR, Erythrocyte Sedimentation Rate; CRP, C-reactive Protein; IL-6, interleukin-6; RF, Rheumatoid Factor; ACPA, Anti-Citrullinated Protein Antibodies; Anti-CarP, Anti-Carbamylated Protein Antibodies.

#### 3.2.5 Janus kinase inhibitors

Janus kinase (JAK) inhibitors are small-molecule targeted synthetic DMARDs that interfere with JAK–STAT intracellular signaling pathways, thereby blocking signal transduction from multiple cytokine receptors involved in rheumatoid arthritis pathogenesis. Because many inflammatory cytokines—including IL-6, interferons, and γ-chain cytokines—signal through JAK-dependent pathways, inhibition of these kinases leads to broad suppression of immune activation and inflammatory responses within the synovium.

Although JAK inhibitors are classified as targeted synthetic DMARDs, they are often discussed alongside biologic DMARDs in biomarker research because they address the same clinical challenge: identifying the most appropriate targeted therapy for individual patients. However, compared with TNF inhibitors or rituximab, the development of validated predictive biomarkers for JAK inhibitor response remains less advanced. Much of the current evidence derives from exploratory studies characterized by relatively small patient cohorts, heterogeneous endpoints, and limited follow-up duration.

Several studies have explored transcriptomic biomarkers associated with response to JAK inhibition. Gene-expression profiling analyses have identified baseline transcriptional signatures that correlate with treatment response to tofacitinib, including pathways related to cellular metabolism, immune activation, and cytokine signaling (102). These findings suggest that pre-existing immune activation states may influence responsiveness to JAK pathway inhibition. However, these transcriptomic signatures have not yet been consistently replicated across independent cohorts, and differences in analytical pipelines and sample processing currently limit their reproducibility.

Real-world observational studies have also attempted to identify clinical and laboratory predictors of response to JAK inhibitors. For example, cohort analyses evaluating baricitinib treatment outcomes have reported associations between baseline disease characteristics, inflammatory markers, and short-term clinical effectiveness (103). However, most of these variables function primarily as prognostic markers, reflecting general disease severity or inflammatory burden, rather than mechanism-specific biomarkers that directly predict responsiveness to JAK pathway inhibition.

More recently, biomarker research has shifted toward integrative modeling approaches, combining multiple molecular and clinical variables to improve predictive accuracy. achine-learning and multi-omics studies have proposed composite models incorporating serum proteomic markers, immune-cell–related features, and clinical parameters to predict response to JAK inhibitors (104). These approaches aim to capture the complexity of cytokine signaling networks affected by JAK inhibition and may ultimately provide more robust predictors than single biomarkers.

Despite these advances, biomarker discovery for JAK inhibitors remains at an early stage. Most candidate predictors require validation in larger prospective studies and must demonstrate reproducibility across different patient populations and analytical platforms. Future work will likely focus on integrating transcriptomic, proteomic, and immunological data to identify patient subgroups whose disease is particularly dependent on JAK-mediated cytokine signaling.

Overall, biomarker research for JAK inhibitors highlights the challenges of predicting response to therapies that affect multiple overlapping cytokine pathways simultaneously. While early studies suggest that baseline immune activation states and multi-parameter molecular signatures may hold promise, clinically applicable predictive biomarkers for JAK inhibitor therapy have not yet been established.

## 4 Translation of biomarkers into clinical practice

### 4.1 Reasons for limited clinical implementation of biomarkers

The integration of biomarkers into routine clinical practice holds substantial promise for improving disease diagnosis, prognostication, and treatment personalization. In rheumatoid arthritis and other chronic immune-mediated diseases, biomarkers have the potential to support early disease stratification, guide therapeutic selection, monitor treatment response, and enhance the efficiency of clinical trials. In the long term, biomarker-informed care may contribute to improved patient outcomes and more cost-effective use of healthcare resources.

However, despite rapid advances in genomics, transcriptomics, proteomics, metabolomics, and digital health technologies, only a small proportion of proposed biomarkers successfully reach clinical implementation. This translational gap reflects multiple interconnected challenges related to reproducibility, cost and accessibility, as well as regulatory and methodological barriers.

#### 4.1.1 Reproducibility, cost and effectiveness

Reproducibility of biomarker findings across independent cohorts and real-world clinical settings remains one of the most critical barriers to implementation. Many biomarkers demonstrate promising sensitivity and specificity in discovery studies but fail to replicate in external validation cohorts or routine practice environments (105). Variability may arise from differences in patient populations, disease stage, treatment exposure, or comorbidities, as well as from inconsistencies in sample collection procedures, assay platforms, and data-analysis pipelines. The broader reproducibility crisis in biomedical research has also been linked to structural factors such as limited funding opportunities for replication studies compared with novel discovery research (106).

Economic considerations further limit the clinical adoption of biomarkers. The development and validation of biomarker assays often require substantial technological infrastructure, including high-throughput sequencing, advanced mass spectrometry platforms, and computational resources for artificial intelligence–based data analysis. As a result, many biomarker tests—particularly those based on multi-omic profiling—remain costly and are not widely available in routine clinical settings. Healthcare systems and insurance providers may be reluctant to reimburse biomarker testing in the absence of robust evidence demonstrating cost-effectiveness and clinical benefit. Moreover, economic evaluation frameworks may struggle to capture the full value of prognostic or predictive biomarkers, particularly when benefits relate to long-term outcomes or treatment optimization (107, 108).

An additional cost-effectiveness challenge is particularly relevant for tissue-based biomarker strategies. Although synovial biopsy and histological assessment can identify broad inflammatory patterns, conventional histology alone is unlikely to provide sufficient resolution for robust treatment selection. The most informative tissue-based biomarkers currently emerge from transcriptomic and multi-omic approaches, including bulk RNA sequencing, single-cell RNA sequencing, spatial transcriptomics, and spatial proteomics. These methods can define synovial pathotypes, fibroblast and macrophage subsets, B-cell niches, tertiary lymphoid structures, and ligand–receptor interaction networks with a level of mechanistic detail that cannot be achieved by routine hematoxylin–eosin staining or basic immunohistochemistry alone.

However, this creates a major translational barrier. RNA-seq-based and spatial-omics approaches remain expensive, require high-quality tissue processing, specialized laboratory infrastructure, bioinformatic expertise, and standardized analytical pipelines. In addition, biopsy site heterogeneity and variability in tissue cellularity may further increase costs by requiring repeated sampling, quality control, or exclusion of non-informative samples. Therefore, although tissue-based biomarkers are among the most promising tools for mechanistic stratification of RA, their direct implementation in routine care may be limited by cost, scalability, and accessibility.

A realistic translational pathway may involve using high-dimensional tissue profiling as a discovery platform to identify simplified biomarker panels that can later be implemented through targeted RNA panels, multiplex immunohistochemistry, digital pathology, or focused spatial-protein assays. In this context, histology may serve as an entry-level screening tool, but clinically useful tissue-based precision medicine will likely require integration with molecular readouts rather than reliance on morphology alone.

Accessibility also represents an important concern. Biomarker testing may be limited by geographical and infrastructural disparities, particularly in rural regions and low- and middle-income countries (LMICs), where access to specialized laboratories and trained personnel is restricted. These inequities risk widening existing gaps in healthcare delivery and may prevent the equitable implementation of precision medicine strategies.

#### 4.1.2 Regulatory and methodological barriers

Regulatory approval processes present additional challenges for biomarker implementation. Unlike pharmaceutical agents, biomarkers may be classified as diagnostic tests, medical devices, or companion diagnostics, resulting in complex and sometimes inconsistent regulatory pathways. Agencies such as the U.S. Food and Drug Administration (FDA), the European Medicines Agency (EMA), and national regulatory bodies must evaluate biomarker performance in terms of analytical validity, clinical validity, and clinical utility. These requirements can lead to prolonged approval timelines and increased development costs.

Regulatory frameworks also differ substantially between regions, and international harmonization remains limited. For example, the introduction of the *In Vitro* Diagnostic Regulation (IVDR) in Europe has increased evidentiary requirements for diagnostic tests, potentially improving patient safety but also creating barriers for smaller laboratories and innovative startups seeking market entry (109). Conversely, in some settings, laboratory-developed tests (LDTs) may operate under less stringent premarket oversight, raising concerns regarding test standardization and quality assurance.

Methodological challenges further complicate biomarker translation. Standardized protocols for biomarker measurement are often lacking, making it difficult to compare results across studies and institutions. Variability in preanalytical processes—including sample collection timing, storage conditions, transportation, anticoagulant choice, centrifugation procedures, and freeze–thaw cycles—can substantially influence biomarker stability and concentration, particularly for labile molecular species. Another critical limitation is the scarcity of certified reference materials and calibration standards, which are essential for ensuring assay accuracy and inter-laboratory comparability.

Currently, many biomarker assays are validated using a “fit-for-purpose” approach, meaning they are optimized for specific research applications such as exploratory screening or mechanistic studies. While this strategy is practical during early biomarker discovery, it may not ensure the analytical robustness and transferability required for diagnostic or prognostic use in clinical practice (110).

#### 4.1.3 Differences between randomized trials and real-world evidence

Despite significant advances in identifying biomarkers capable of predicting response to biological therapy, including in rheumatoid arthritis, their clinical implementation remains limited. In randomized controlled trials (RCTs), biomarkers demonstrate convincing prognostic ability (111) However, in real clinical practice, this may not be confirmed, as patients often have multiple comorbidities, receive combination therapy, and laboratory measurements are performed irregularly, using different techniques and reagents. All of this introduces significant variability into the data and makes it impossible to reproduce the exact threshold values derived in the RCTs (112).

The differences between RCTs and real-world data (RWD) are compounded by the fact that real patients are not a homogeneous cohort, but a heterogeneous population in which response to therapy also depends on socioeconomic and behavioral factors. Patients may not attend doctor’s appointments, miss doses of medication, face difficulties accessing laboratory services, or experience depression, which affects their perception of pain and assessment of disease activity. Under these conditions, the biomarker value may be distorted by non-compliance with therapy or concomitant inflammation from obesity or infection (113).

As a result, there is a gap between the scientific potential of biomarkers and their actual impact on clinical decision-making. To bridge this gap, it is necessary not only to validate biomarkers in new cohorts, but also to integrate their use into clinical workflows.

## 5 Future directions

A recurring theme across virtually all biomarker classes is the discrepancy between biological plausibility and clinical utility. Biomarkers such as MTX polyglutamates, calprotectin, transcriptomic signatures, and synovial pathotypes are supported by strong mechanistic rationale, yet few have demonstrated sufficient reproducibility, standardization, and prospective clinical benefit to justify routine implementation. In contrast, biomarkers with the greatest current clinical utility—such as drug concentrations and anti-drug antibodies—often provide limited mechanistic insight but directly support therapeutic decision-making. This tension highlights the central challenge of RA precision medicine: translating biologically informative biomarkers into scalable and actionable clinical tools. (**Table 4**).

**Table 4:** Evidence grading and translational readiness of RA biomarkers.

Biomarker/biomarker class	Main biological level	Strength of evidence	Level of validation	Reproducibility	Translation potential	Interpretation
RF and ACPA	Serological autoimmunity	High for diagnosis/prognosis; moderate for treatment prediction	Validated in large cohorts and clinical practice	High	High for stratification; limited for drug selection	RF and ACPA are robust clinical-grade biomarkers for diagnosis, prognosis, and disease stratification. However, their ability to predict response to specific therapies is limited, except in selected contexts such as improved response to rituximab or abatacept in seropositive RA (39).
CRP, ESR, DAS28, SDAI	Systemic inflammation/clinical disease activity	High for monitoring disease activity; low-to-moderate for predicting mechanism-specific response	Widely validated clinically	High for monitoring, lower for prediction	High for routine monitoring	These markers are clinically useful but biologically non-specific. They reflect systemic inflammatory burden rather than dominant synovial mechanisms and may be misleading under therapies that directly suppress acute-phase reactants, especially IL-6 blockade and JAK inhibition (22).
Methotrexate polyglutamates	Pharmacological exposure	Moderate	Replicated in several cohorts, but thresholds not standardized	Moderate	Moderate	MTX polyglutamates are mechanistically plausible because they reflect intracellular drug exposure. However, erythrocyte MTX-PGs are indirect surrogates of drug activity in immune or synovial cells, and clinically actionable thresholds remain insufficiently standardized. Current utility is greater for exposure assessment than for routine treatment selection (60).
Calprotectin	Myeloid inflammation	Moderate	Replicated in observational studies; limited prospective validation	Moderate	Moderate	Calprotectin is biologically attractive because it reflects myeloid-cell activation more directly than hepatic acute-phase proteins. It may improve monitoring of synovitis and inflammatory burden, but clinical cutoffs, assay standardization, and incremental value over composite indices remain unresolved (26).
Imaging biomarkers: power Doppler ultrasound, SMI, MRI	Tissue inflammation/vascularity/structural damage	Moderate-to-high for detecting residual inflammation; moderate for prediction	Validated for inflammation detection; less validated for treatment selection	Moderate, operator- and protocol-dependent	Moderate-to-high for monitoring; lower for drug selection	Power Doppler and MRI identify subclinical synovitis and residual inflammation better than clinical examination. However, their predictive value for choosing a specific therapeutic mechanism remains limited by operator dependence, scoring variability, cost, and lack of universally accepted thresholds.
TNF inhibitor-associated soluble biomarkers, including CXCL10/CXCL13	Cytokine and chemokine networks	Low-to-moderate	Partial validation in selected cohorts	Low-to-moderate	Moderate if incorporated into panels	Chemokines such as CXCL10 and CXCL13 are mechanistically linked to immune-cell recruitment and inflammatory organization, but single-analyte prediction of TNF inhibitor response remains inconsistent. Their future value is more likely within composite molecular or tissue-informed panels than as standalone tests (94).
IL6R polymorphisms and IL-6 pathway markers	Cytokine signaling/pharmacogenetics	Low-to-moderate	Limited and heterogeneous validation	Low-to-moderate	Moderate	IL-6 pathway biomarkers are mechanistically relevant for tocilizumab response, but genetic effects are modest and inconsistent across populations. Suppression of CRP after IL-6 blockade is a pharmacodynamic marker, not necessarily a reliable indicator of synovial resolution (100).
ACPA status, IFN signature, CTLA4 variants for abatacept response	Adaptive immunity/T-cell costimulation	Moderate for ACPA; low for IFN and CTLA4 markers	ACPA associations replicated; molecular markers need validation	Moderate for ACPA, low-to-moderate for others	Moderate	ACPA positivity is one of the more reproducible clinical predictors of abatacept response, consistent with the drug’s mechanism of T-cell costimulation blockade. IFN signatures and CTLA4 variants are biologically plausible but remain investigational (8, 101).
Rituximab-associated synovial B-cell signatures	Tissue humoral immunity	Moderate-to-high	Supported by biopsy-driven studies, including R4RA	Moderate	High conceptually; moderate clinically	Synovial B-cell-rich signatures provide one of the strongest examples of mechanism-aligned tissue biomarkers. They directly reflect local target abundance and humoral immune organization. However, implementation requires biopsy access, standardized molecular assays, and prospective treatment-selection trials (6, 65).
BCR repertoire features after rituximab	Adaptive immune repertoire	Low-to-moderate	Small cohorts; biologically informative	Low-to-moderate	Moderate for monitoring; exploratory for selection	Persistence of dominant B-cell clones or patterns of naïve B-cell repopulation may reflect incomplete immune reset or subsequent response. These data provide mechanistic insight into rituximab response and relapse, but sample sizes remain small and clinical thresholds are not defined (97, 98).
Synovial pathotypes	Tissue immunophenotype	Moderate-to-high	Replicated in biopsy cohorts; prospective therapeutic validation emerging	Moderate	High	Synovial pathotypes integrate multiple cellular and molecular features of the target tissue. Lympho-myeloid, diffuse-myeloid, and pauci-immune/fibroblastic patterns provide a biologically coherent framework for explaining response and resistance. Their main limitation is the need for standardized biopsy processing and clinically deployable classification algorithms (63, 64).
Fibroblast subsets	Stromal tissue biology	Moderate mechanistic evidence; low clinical validation	Strong experimental and single-cell evidence; limited prospective clinical validation	Moderate in discovery studies	High future potential	Fibroblast subsets define stromal programs linked to inflammation, tissue destruction, and multidrug resistance. Fibroblast-dominant or pauci-immune synovitis may explain poor response to several immune-targeted biologics. However, fibroblast biomarkers are not yet clinically actionable and require simplified assays and prospective validation (67, 68).
Macrophage subsets, including MerTK+ and MerTK− states	Innate tissue immunity	Moderate	Supported by single-cell and tissue studies; limited interventional validation	Moderate	High for remission assessment and flare prediction	Macrophage states may distinguish active inflammatory synovitis from tissue-level remission. MerTK+ macrophages are associated with resolution programs, whereas MerTK− inflammatory macrophages mark active disease. These markers are promising for assessing remission depth and flare risk, but are not yet routine clinical tools (70).
B-cell niches and tertiary lymphoid structures	Spatial humoral immunity	Moderate mechanistic evidence	Histological and molecular support; limited prospective validation	Moderate	High for mechanism-based stratification	B-cell niches and TLS indicate organized local adaptive immunity, CXCL13-driven recruitment, and potential local autoantibody production. They may improve prediction of response to B-cell depletion or costimulation blockade, but bulk B-cell signatures must be distinguished from spatially organized TLS (116).
scRNA-seq	High-resolution cellular profiling	High discovery value; low clinical readiness	Strong discovery validation; limited clinical implementation	Moderate across platforms	High future potential	scRNA-seq has transformed understanding of RA synovial cell states, including pathogenic fibroblast, macrophage, B-cell, and T-cell subsets. Its current value is primarily to define simplified biomarkers for translation rather than to function as a routine clinical assay (68).
Spatial transcriptomics	Tissue architecture/spatial gene expression	Moderate discovery evidence; low clinical readiness	Emerging validation	Low-to-moderate	High future potential	Spatial transcriptomics preserves tissue architecture and can define pathogenic niches, such as B-cell aggregates, macrophage–fibroblast interfaces, and perivascular immune zones. Its main limitation is cost, platform variability, and the need for clinically interpretable spatial metrics (117).
Spatial proteomics and multiplex imaging	Protein-level tissue organization	Exploratory-to-moderate	Early-stage studies	Low-to-moderate	High future potential	Spatial proteomics may bridge discovery omics and diagnostic pathology by measuring therapeutic targets and cell neighborhoods directly at the protein level. However, standardization of antibody panels, image analysis, and cross-center reproducibility remains a major challenge.

A consistent message across recent RA biomarker literature is that single-analyte predictors rarely generalize, as RA is driven by heterogeneous and dynamic immune circuits across patients and over time. Therefore, a key future direction is the transition from isolated biomarkers to mechanism-aligned, system-level stratification frameworks that can be prospectively validated and implemented using scalable assays.

A major conceptual advance is the shift toward defining patient-level immunotypes—coordinated patterns of immune cell states and functional programs—rather than relying on individual cytokines or autoantibodies. Such higher-order immune configurations may improve both disease classification and prediction of therapeutic response, particularly when integrated with clinical data. This favors composite immune profiling approaches (e.g., flow cytometry, CyTOF, targeted transcriptomics, serum protein panels) that can be standardized across centers (114).

In parallel, there is a growing focus on integrative, tissue-informed biomarkers that reflect the functional state of the synovial immune microenvironment. These approaches incorporate immune cell phenotypes, activation states, and TCR/BCR repertoires, capturing antigen specificity and clonal dynamics. Within this framework, biomarkers are increasingly viewed as multi-layered descriptors of pathogenic immune circuits rather than isolated measurements.

Emerging therapeutic strategies, including CAR-T–based approaches, further reinforce this paradigm. Early studies suggest that B-cell–targeted CAR-T therapies can induce deep immune reset, and predictive biomarkers in this context may include baseline clonal architecture, antigen-specific receptor features, and patterns of immune reconstitution. This underscores the importance of high-resolution immune monitoring, including repertoire sequencing and single-cell profiling, for both current and next-generation therapies (115).

The strongest biologically coherent predictors of treatment response increasingly arise from synovial tissue analysis. RNA-seq studies have identified distinct synovial pathotypes associated with disease progression and treatment outcomes, while the biopsy-driven R4RA trial demonstrated that synovial molecular signatures can guide mechanism-based therapy selection (6, 64). Similarly, immune repertoire studies in rituximab-treated patients indicate that persistence of dominant BCR clones and patterns of B-cell repopulation are associated with clinical outcomes (97, 98).

Together, these findings support a shift toward biomarker-guided interventional strategies, in which biologically defined endotypes are matched to targeted therapies and validated in prospective settings.

Importantly, while recent reviews have described RA heterogeneity and multi-omics landscapes, the present review specifically emphasizes mechanistically grounded biomarkers of therapeutic response, with a focus on cellular interactions, immune circuits, and tissue-level processes. By integrating pharmacological, molecular, and synovial biology data, this work aims to bridge the gap between descriptive biomarker discovery and clinically actionable precision medicine.

## 6 Conclusion

Rheumatoid arthritis is increasingly understood as a collection of partially overlapping pathogenic endotypes rather than a single uniform inflammatory disease. This explains why clinically similar patients may differ markedly in response to the same DMARD. The central challenge for precision medicine is therefore not simply to identify additional biomarkers, but to integrate them into a clinically usable framework that links measurable signals to dominant pathogenic mechanisms within the synovium.

A future biomarker framework for RA should combine several complementary layers. The first layer includes conventional clinical and serological markers, such as disease activity indices, RF, ACPA, CRP, ESR, drug concentrations, and anti-drug antibodies. These remain essential for diagnosis, prognosis, monitoring, and recognition of pharmacokinetic failure. However, they are insufficient to define the tissue mechanism driving treatment response or resistance. The second layer should include molecular and multiomic biomarkers, including transcriptomic, proteomic, metabolomic, genomic, epigenomic, and immune-repertoire data. These approaches can identify pathway-level activity, immune-cell activation states, and molecular signatures associated with response to TNF inhibitors, rituximab, tocilizumab, abatacept, JAK inhibitors, and methotrexate.

The third and most mechanistically informative layer is synovial tissue immunophenotyping. Synovial pathotypes, fibroblast subsets, macrophage states, B-cell niches, tertiary lymphoid structures, and ligand–receptor interaction networks provide direct information about the cellular circuits operating in the target tissue. In this model, lympho-myeloid or B-cell-rich synovitis may support B-cell-targeted or costimulation-directed strategies, myeloid-rich inflammation may indicate cytokine-dominant disease, whereas pauci-immune or fibroblast-dominant synovitis may explain poor response to several immune-targeted biologics. Thus, synovial-endotype-driven therapy represents a logical next step beyond empirical treatment escalation.

Artificial intelligence and machine learning will be essential for converting these heterogeneous data types into clinically interpretable prediction tools. Rather than replacing biological reasoning, AI-based models should integrate clinical variables, pharmacological data, serum biomarkers, omics profiles, synovial histology, and spatial features into transparent classifiers of therapeutic response. Such classifiers should aim not only to predict “response” or “non-response,” but to assign patients to biologically meaningful treatment pathways, such as conventional therapy, biologic escalation, JAK inhibition, B-cell depletion, IL-6 blockade, or alternative strategies for stromal-dominant disease.

Integrated biomarker panels are therefore likely to be more useful than single markers. A practical RA precision-medicine panel may include: clinical disease activity, autoantibody status, inflammatory proteins, drug exposure and immunogenicity markers, selected transcriptomic or proteomic modules, and tissue-derived endotype features. Digital pathology may become a key translational bridge by converting synovial biopsies into standardized quantitative readouts of cellular density, lymphoid organization, macrophage–fibroblast architecture, and stromal activation. Combined with spatial transcriptomics, spatial proteomics, and single-cell-derived reference maps, digital pathology could make tissue immunophenotyping more scalable and reproducible.

Clinical implementation will require prospective multicenter biomarker-guided trials, harmonized biopsy protocols, standardized omics and imaging pipelines, external validation of machine learning models, and demonstration of cost-effectiveness. Importantly, predictive models must be interpretable, clinically actionable, and compatible with routine rheumatology workflows. The goal is not to replace treat-to-target principles, but to make them biologically informed: selecting the most appropriate therapeutic mechanism earlier, reducing trial-and-error prescribing, and improving the durability of response.

In conclusion, the next stage of RA biomarker development should shift from isolated circulating analytes toward integrated, synovial-endotype-driven prediction models. By combining clinical assessment, pharmacological monitoring, multiomic profiling, digital pathology, and tissue immunophenotyping, precision medicine in RA may move from conceptual promise to therapeutic decision-making grounded in the biology of the individual patient’s disease.
